# Bidirectional CRISPR screens decode a GLIS3-dependent fibrotic cell circuit

**DOI:** 10.1038/s41586-025-09907-x

**Published:** 2026-01-07

**Authors:** Vladislav Pokatayev, Alok Jaiswal, Angela R. Shih, Åsa Segerstolpe, Bihua Li, Elizabeth A. Creasey, Yanhua Zhao, Crystal Lin, Shane Murphy, Chih-Hung Chou, Daniel B. Graham, Ramnik J. Xavier

**Affiliations:** 1 Center for Computational and Integrative Biology, Massachusetts General Hospital, Harvard Medical School, Boston, MA USA; 2 Department of Molecular Biology, Massachusetts General Hospital, Harvard Medical School, Boston, MA USA; 3 Broad Institute of MIT and Harvard, Cambridge, MA USA; 4 Department of Pathology, Massachusetts General Hospital, Harvard Medical School, Boston, MA USA; 5 Klarman Cell Observatory, Broad Institute of MIT and Harvard, Cambridge, MA USA; 6 Center for the Study of Inflammatory Bowel Disease, Massachusetts General Hospital, Boston, MA USA

**Keywords:** Mechanisms of disease, Cell signalling, Scale invariance, Immunology, Systems analysis

## Abstract

The stromal cell compartment plays a central part in the maintenance of tissue homeostasis by coordinating with the immune system throughout inception, amplification and resolution of inflammation^1^. Chronic inflammation can impede the phased regulation of tissue restitution, resulting in the scarring complication of fibrosis. In inflammatory bowel disease, stromal fibroblasts have been implicated in treatment-refractory disease and fibrosis^2,3^; however, their mechanisms of activation have remained undefined. Through integrative single-cell and spatial profiling of intestinal tissues from patients with inflammatory bowel disease, we uncovered a pathological cell nexus centred on inflammation-associated fibroblasts. These fibroblasts were induced by proinflammatory macrophages (*FCN1*^+^*IL1B*^+^) and, in turn, produced profibrotic cytokine IL-11. We investigated the inflammation-associated fibroblast activation program at a mechanistic level using genome-wide CRISPR knockout and activation screens and identified the transcription factor GLIS3 as a key regulator of a gene regulatory network governing expression of inflammatory and fibrotic genes. We further demonstrated that the magnitude of the GLIS3 gene expression program in intestinal biopsies could be used to stratify patients with ulcerative colitis by disease severity, and that fibroblast-specific deletion of *Glis3* in mice alleviated pathological features of chronic colitis. Taken together, our findings identify a critical immune–stromal cell circuit that functions as a central node in the inflammation–fibrosis cycle.

## Abstract (web summary)

Integrated single-cell and spatial data analysis, combined with bidirectional CRISPR screens, identify the transcription factor GLIS3 as a key driver of chronic inflammation and fibrosis and a potential marker of disease severity in patients with ulcerative colitis.

## Main

Chronic inflammation overstimulates fibrogenesis, culminating in fibrosis, a condition that accounts for 45% of disease-related deaths and has limited treatment options^1^. Single-cell profiling has identified functionally distinct and location-specific fibroblasts as central drivers of fibrosis^4^. In addition to pan-tissue *PI16*^+^ or *COL15A1*^+^ fibroblasts, inflammation-associated signals instigate fibroblast states that impair the resolution of injury-induced inflammation, deposit excessive fibrotic collagen and alter tissue mechanics^5–7^. In several human inflammatory diseases, *CXCL10*^+^*CCL19*^+^ and *SPARC*^+^*COL3A1*^+^ fibroblasts expand in immune- or vasculature-associated tissue niches, respectively, in which they promote pathology^8^. Targeting these processes is challenging owing to our limited understanding of the molecular basis of disease-associated fibroblasts and the fact that existing immunosuppressives block proinflammatory mediators, which are not fibroblast-specific.

We previously reported that inflammation-associated fibroblasts (IAFs) were expanded in inflammatory bowel disease (IBD) and expressed an inflammatory and fibrogenic gene signature associated with resistance to anti-tumour necrosis factor (TNF) therapy^2,3^. This signature was enriched before treatment resistance, implying that IAFs promote disease progression despite medical intervention^9^. RNA sequencing (RNA-seq) has also implicated oncostatin M (OSM) signalling and neutrophil recruitment in therapy resistance; however, the IAF-specific determinants and their relation to other resistance markers are not fully understood^10,11^. Identifying the underlying cellular and molecular wiring of fibroinflammatory processes will be critical to the development of new treatments, as general immunosuppressives do not improve long-term fibrotic outcomes, and biologics can have adverse effects or lose efficacy^12^. As fibroblasts have emerged as central drivers of tissue remodelling after inflammation, we aimed to decipher the inter- and intracellular wiring of IAFs in a disease marked by fibrosis development.

## A single-cell and spatial atlas of IBD

**Fig. 1:**
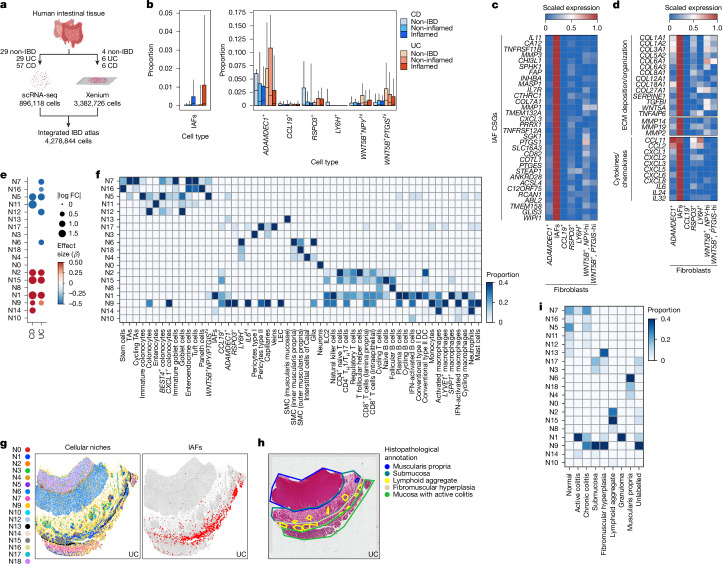
An integrated single-cell and spatial atlas reveals inducible IAFs in pathological cellular niches. **a**, Schematic of the integrated IBD atlas workflow using scRNA-seq or Xenium to profile non-IBD individuals and patients with CD or UC. **b**, Changes in proportions of fibroblasts stratified by disease. Box plots represent quartiles, with medians as the centre and whiskers the 10–90% range. Statistics were analysed using scCODA (Bayesian Dirichlet multinomial model) with smooth muscle cells as reference (false discovery rate (FDR) < 20%) (Methods). Numbers of samples for each category: non-IBD, 29; CD inflamed, 28; CD non-inflamed, 54; UC inflamed, 25; UC non-inflamed, 22. **c**, Pseudobulk expression heatmap of scaled average IAF-specific genes (Wilcoxon signed-rank test (two-sided), *P* < 0.05; log fold change > 3; expression in more than 25% of IAFs, fewer than 10% of non-IAFs). **d**, Pseudobulk scaled expression heatmap of IAF genes involved in ECM deposition and organization or in cytokine and chemokine production. **e**, Dot plot showing effect size (*β*) and absolute log_2_ fold change (FC) for niche enrichment across CD and UC compared with non-IBD samples. Blue indicates enrichment in non-IBD samples and red in CD and UC samples. scCODA with N3 reference niche was used for the analysis. FDR < 20% for niche change in abundance. **f**, Heatmap of statistically enriched cell type proportion abundance across niches. Chi-squared test with *P* < 0.05 as the significance threshold. **g**, Left, visualization of cellular niches projected on to a Xenium-profiled UC tissue section. Right, distribution of IAFs on the same tissue section, showing dense distribution in niche N1. **h**, Haematoxylin and eosin (H&E) section of UC tissue from **g** showing annotated anatomical and pathological tissue regions. Images are representative of the sample cohort; *n* = 16 patients. **i**, Heatmap depicting the enriched niches within anatomical and pathological tissue domains. CSGs, cell-specific genes; DC, dendritic cell; ILC2, type 2 innate lymphoid cell; LEC, lymphatic endothelial cell; SMC, smooth muscle cell; TA, transit-amplifying cell; T_H_1 cell, T helper 1 cell. Illustrations in **a** were created using BioRender. Pokatayev, V. (2025) https://BioRender.com/gnpbg27.

**Extended Data Fig. 1:**
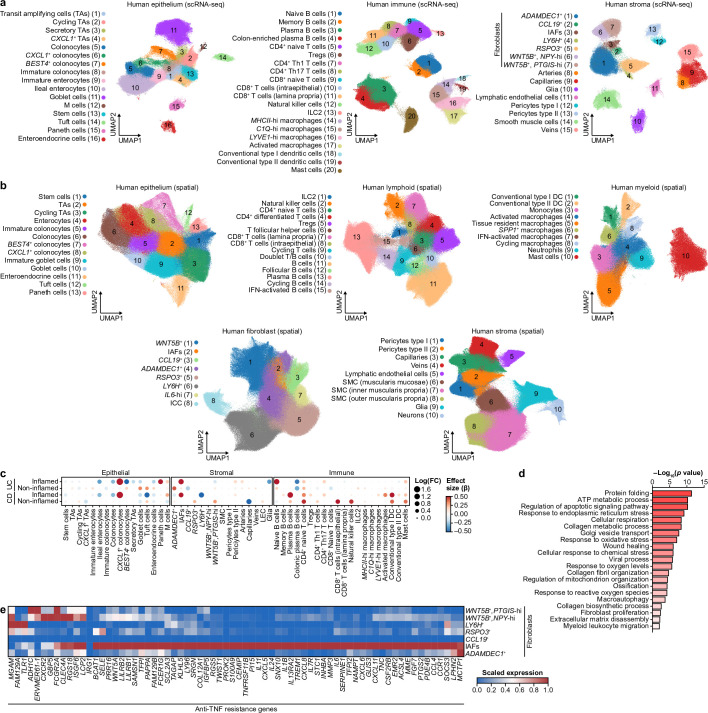
An integrated single-cell and spatial atlas reveals inducible, inflammation-associated fibroblasts. (**a**) UMAP of epithelial, immune, and stromal cell compartments from profiling IBD patients with scRNA-seq. (**b**) UMAP of epithelial, lymphoid, myeloid, fibroblast, and stromal cell compartments from profiling IBD patients with Xenium-based spatial transcriptomics. (**c**) Dot plot showing effect size (*β*) and absolute log_2_ fold change (logFC) for cell type enrichment across CD and UC compared to non-IBD controls. Blue indicates enrichment in non-IBD and red indicates enrichment in CD and UC. Analysis was done using *scCODA* with the SMC cells as the reference. Only cell types that passed the significance threshold (FDR < 20%) were considered changed in abundance. See Methods for more details. (**d**) Pathway enrichment analysis of IAF-specific genes. Benjamini-Hochberg adjusted *P* values from a hypergeometric test (one-sided) for enriched pathways were plotted. See Methods for details. (**e**) Pseudobulk scaled expression heatmap of anti-TNF resistance signature^21^ plotted across all fibroblast subtypes in the integrated IBD atlas.

To systematically decipher the shared and distinct cellular and molecular drivers of Crohn’s disease (CD) and ulcerative colitis (UC), the principal clinical subtypes of IBD, we integrated single-cell RNA-seq (scRNA-seq) data from the small and large intestine with publicly available IBD datasets^2,9,11,13^ to construct a single-cell IBD atlas comprising 29 non-IBD control samples, 29 samples from patients with UC and 57 samples from patients with CD (Fig. 1a and Extended Data Fig. 1a). To contextualize cellular spatial relationships and their associations with distinct histopathological features, we applied Xenium-based single-cell spatial profiling and mapped these cells in the intestinal tissue. We included pathologist-annotated tissue resections from the small or large intestine of four non-IBD control samples (normal cuff of colon adjacent to diverticulitis), three ileal and three colonic samples from patients with CD, and six colonic samples from patients with UC (Supplementary Data 1). These atlases profile more than four million intestinal cells across epithelial, immune, stromal and fibroblast compartments and provide a comprehensive framework for study of the aetiology of IBD (Fig. 1a and Extended Data Fig. 1b).

Across IBD samples, we observed significant changes in cell type abundance within defined intestinal compartments (Extended Data Fig. 1c). In the fibroblast compartment, most cells did not show any change in abundance between diseases, with the exception of reparative *ADAMDEC1*^+^ fibroblasts^14^, which decreased in abundance in inflamed CD and UC, and a subset of *IL11*-expressing IAFs that was expanded in inflamed CD and UC (Fig. 1b). IL-11 is a constituent of the IL-6 family, a group of cytokines that dictate the balance between tissue repair and fibrosis, with IL-11 having emerged as a profibrotic cytokine^15^. In addition to *IL11*, IAFs specifically expressed genes associated with fibrosis and impaired tissue functionality, including *CD82*, *PRRX1* and *CHI3L1* (refs. ^16–18^) (Fig. 1c). IAFs also upregulated extracellular matrix (ECM) remodelling genes, including *COL1A1* and *COL6A1*, as well as *ABL2*, which drives cytoskeletal rearrangement^19,20^ (Fig. 1c,d). Furthermore, IAFs expressed neutrophil-recruiting chemokines (*CXCL3*, *CXCL5*, *CXCL8*), indicating that they may direct immune cell activity during disease^9,11^ (Fig. 1d). Pathway analysis of the IAF transcriptome demonstrated enrichment of ECM organization alongside inflammatory gene expression (Extended Data Fig. 1d). We previously reported that several IAF genes are associated with a signature or refractory response to anti-TNF treatment^2,21^ (Extended Data Fig. 1e).

**Extended Data Fig. 2:**
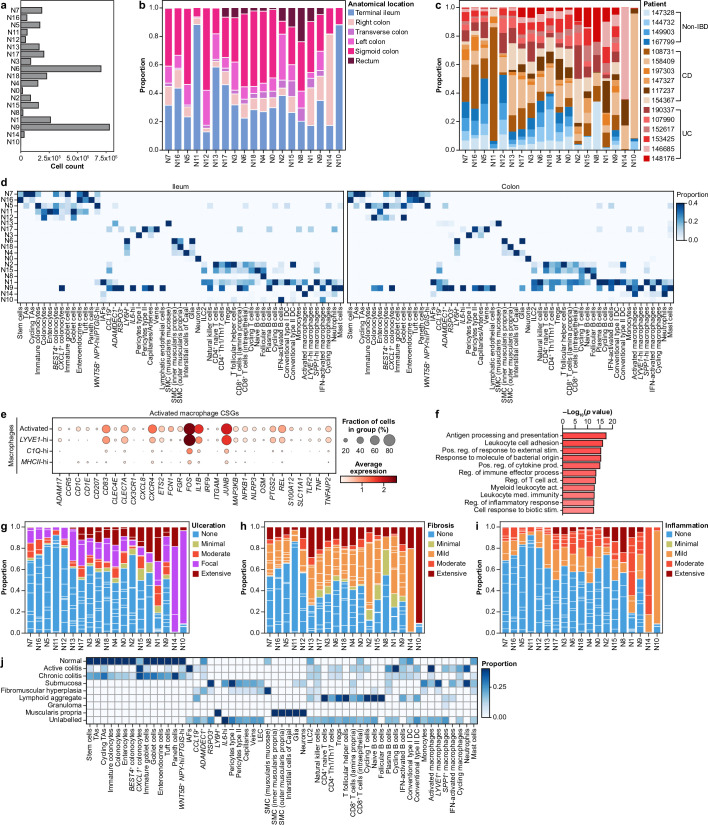
An integrated single-cell and spatial atlas reveals that inflammation-associated fibroblasts reside in pathological cellular niches. (**a**) Sum of cell counts of each niche across all patients profiled in the spatial atlas. (**b**) Barplot depicting relative proportion of each niche along the gastrointestinal tract. (**c**) Barplot depicting relative proportion of each niche across the 16 patients profiled. (**d**) Heatmap depicting the proportion of cell type abundance across ileal (n = 3) or colonic (n = 13) niches. Only statistically enriched cell types are plotted (Chi-squared two-sided test, Bonferroni adjusted *P* < 0.05). (**e**) Expression dot plot of selected immune genes from activated macrophages. (**f**) Pathway enrichment analysis of activated macrophage-specific genes. Benjamini-Hochberg adjusted *P* values from a hypergeometric test (one-sided) for enriched pathways were plotted. (**g**) Barplot depicting relative proportion of each niche across qualitative grades of tissue ulceration. (**h**) Barplot depicting relative proportion of each niche across qualitative grades of tissue fibrosis. (**i**) Barplot depicting relative proportion of each niche across qualitative grades of tissue inflammation. (**j**) Heatmap of cell type proportions across distinct anatomical and histopathological tissue domains as defined by a pathologist on all human patient tissues profiled. Only statistically enriched cell types are plotted (Chi-squared two-sided test, Bonferroni adjusted *P* < 0.05).

Given the increased prevalence of IAFs in patients with inflamed UC or CD, we proposed that their expansion could reflect concurrent intestinal tissue remodelling. We defined IAF-anchored multicellular niches by implementing cell neighbourhood analysis, systematically quantifying cellular neighbours within a 30-µm radius to capture contact-ligand–receptor and secreted-ligand–receptor interactions^22^. We identified 19 distinct cellular niches, each comprising 5,298 to 778,470 cells summed across all samples (Extended Data Fig. 2a). The niches corresponded to distinct anatomical layers, and several showed significant changes in abundance between diseases (Extended Data Fig. 2b,c). To account for anatomical variation, we examined niche–cell-type composition separately in colonic and ileal samples. Whereas niche compositions remained broadly consistent between anatomical sites, several epithelial cell niches (N5, N11, N12) showed greater compositional variation (Extended Data Fig. 2d). In CD and UC, mucosal epithelial niches (N7, N16, N5, N11, N12) were depleted; conversely, lymphocyte-enriched (N2 and N15) and myeloid-enriched (N1, N9, N14) niches were significantly expanded (Fig. 1e,f). IAFs were statistically enriched in niches N1 and N14 in both colon and ileum; these comprised stromal and immune cells, with the cell type showing the strongest shared enrichment being *FCN1*^+^*IL1B*^+^ macrophages (activated macrophages), which resemble CD68^+^ infiltrated mucosal macrophages in IBD^23^ (Fig. 1f and Extended Data Fig. 2e). These monocyte-derived macrophages were characterized by canonical activation pathways and specifically expressed proinflammatory cytokines (*IL1B*, *TNF*, *OSM*) and innate immune sensors (*TLR2*, *NLRP3*), indicating that N1 and N14 mark inflamed regions influenced by stromal–immune interactions (Extended Data Fig. 2e,f). Histological characterization suggested that N1 and N14 were highly prevalent in tissues in advanced stages of fibrosis and ulceration (Extended Data Fig. 2g–i). We then refined our analyses to map the specific histopathological regions associated with these niches, observing that N1 and N14 were localized to mucosal regions with active or chronic colitis (Fig. 1g–i and Extended Data Fig. 2j). Collectively, these results showed that IAFs localize to niches associated with active disease, potentially integrating signals from the tissue microenvironment to enact fibroinflammatory programs associated with disease.

## An IL-11 cell circuit governs fibrosis

**Extended Data Fig. 3:**
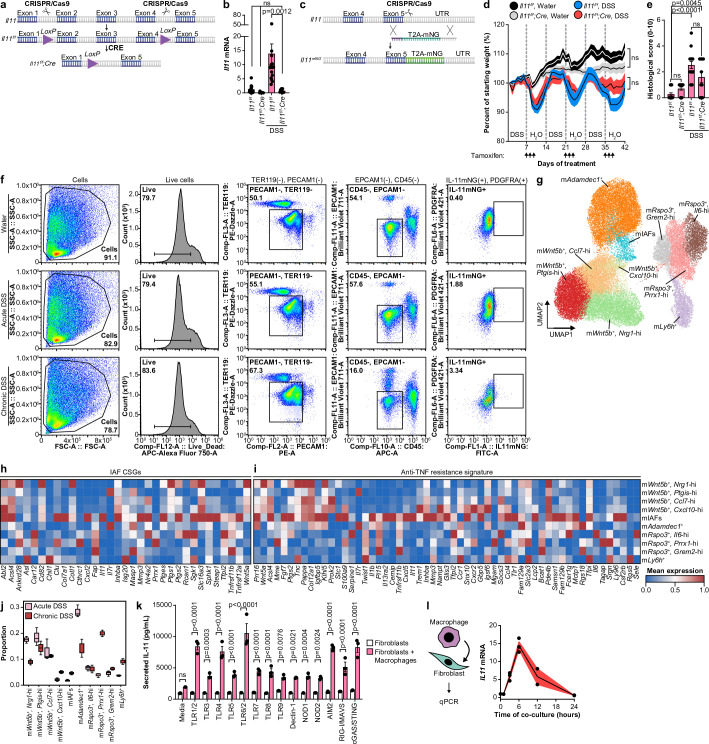
Pro-fibrotic IAFs secrete IL-11 in response to activated macrophages. (**a**) Schematic of *Il11*^*f/f*^ mice generated by flanking exons 2–4 with *LoxP* for Cre-mediated excision. (**b**) qPCR quantification of *Il11* from tissue lysates from Fig. 2a, normalized to *Eef2*. (**c**) Schematic of *mNeonGreen* knock-in at the *Il11* terminus with homology-directed repair. (**d**) Percentage of starting weight of mice from Fig. 2a (see Methods for treatment). Filled lines represent s.e.m. Two-way repeated measures ANOVA. (**e**) Histopathological scoring (see Methods) on H&E-stained tissues from Fig. 2a. (**f**) Gating strategy to quantify IL-11^mNG^ after water or DSS treatment. Plots are representative of the sample cohort. Related to Fig. 2e, g. (**g**) UMAP of PDGFRA+ fibroblasts after acute or chronic DSS. (**h**) Pseudobulk scaled expression heatmap of human IAF genes across PDGFRA+ fibroblasts after acute or chronic DSS. (**i**) Pseudobulk scaled expression heatmap of human anti-TNF resistance genes across PDGFRA+ fibroblasts after acute or chronic DSS. (**j**) Proportion changes of PDGFRA+ fibroblasts across DSS models. Box plots represent the quartiles with medians as the center, and whiskers the 10–90% range. Number of samples for each category: Acute DSS = 3; Chronic DSS = 2. (**k**) Secreted IL-11 measured from co-cultures of ligand-activated monocyte-derived macrophages (see Methods for concentrations) and primary human colon fibroblasts. n = 3 cell lines per condition. (**l**) qPCR quantification of *IL11* from fibroblasts co-cultured with TLR2/6-activated macrophages over time, normalized to *HPRT*. Filled lines represent s.e.m. n = 3, except at 12 h where n = 2 cell lines. All mice were co-housed. Unless otherwise stated, statistics are by a two-way ANOVA with Tukey’s multiple comparison test on distinct biological replicates and error bars are mean ± s.e.m. ns, not significant. Illustrations in **a**, **c** and **l** were created with BioRender (https://biorender.com). Source Data

To define the spatial roles of IAFs, we generated conditional *Il11*-knockout mice alongside a reporter line, enabling elucidation of IAF function and distribution in a colitis model with stromal-driven fibrosis. We flanked *Il11* exons 2–4 with *loxP* sites (*Il11*^*f/f*^) and bred this line on to tamoxifen-inducible *cre/ERT2*, enabling temporal deletion of *Il11* (*Il11*^*f/f*^*;cre*) (Extended Data Fig. 3a,b). We also engineered a novel *Il11* reporter mouse by inserting T2A-mNeonGreen (mNG) before the *Il11* stop codon (*Il11*^*mNG*^), thereby leveraging selective expression of *IL11* in IAFs (Extended Data Fig. 3c).

**Fig. 2:**
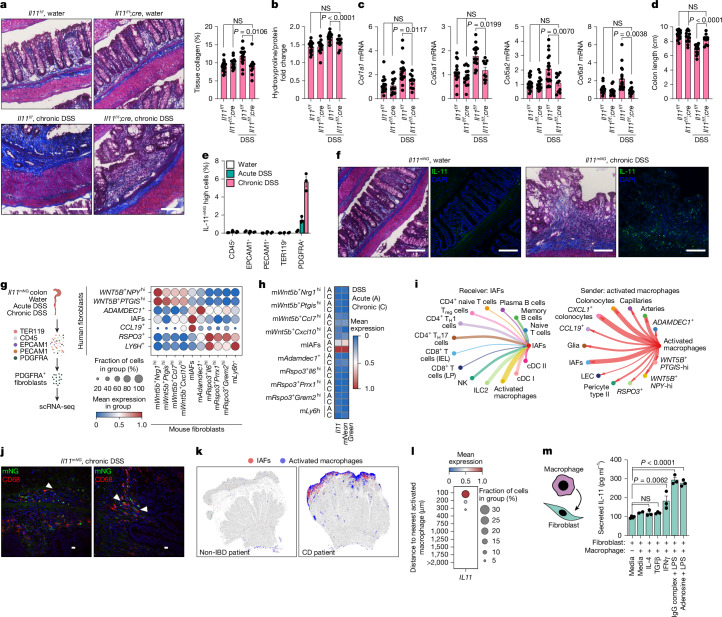
An IL-11 cell circuit governs fibrosis. **a**, Masson’s trichrome-stained *Il11*^*f/f*^ and *Il11*^*f/f*^*;cre* colons (8–18 weeks) treated with water or chronic DSS (left). Total collagen percentage from three pooled experiments (right). *Il11*^*f/f*^-water, *n* = 10; *Il11*^*f/f*^*;cre*-water, *n* = 14; *Il11*^*f/f*^-DSS, *n* = 17; *Il11*^*f/f*^*;cre-*DSS, *n* = 10 mice. **b**, Colonic hydroxyproline normalized to total protein for tissues from **a**. **c**, qPCR quantification of collagens normalized to *Eef2* from tissues from **a**. **d**, Colon length measurements from **a**. **e**, Percentage of IL-11^mNG^ cells across lineages after the indicated treatments. Water-treated, *n* = 2; DSS-treated, *n* = 3 mice. **f**, Masson’s trichrome-stained (left) and immunofluorescence-stained (right) *Il11*^*mNG*^ tissues after DSS. Images are representative of three independent experiments. **g**, Schematic of PDGFRA^+^ fibroblast isolation from acute and chronic DSS-treated *Il11*^*mNG*^ mice (8–14 weeks) (left). Dot plot mapping human fibroblast gene signatures across mouse fibroblasts (right). **h**, Pseudobulk expression heatmap depicting scaled average expression of *Il11* and *mNeonGreen* from acute and chronic DSS treatment groups. **i**, Spatial niche-aware probability of intercellular communication. Edge thickness or node size depicts communication strength. Significant signals received by IAFs (left) and sent from activated macrophages (right). **j**, Immunofluorescence of chronic DSS-treated colons from *Il11*^*mNG*^ mice depicting proximal macrophage (CD68, red) and IL-11^mNG^ fibroblast (green) localization. Arrowheads indicate signal adjacency. Images are representative of three independent experiments. **k**, Spatial projection of IAFs and activated macrophages in non-IBD and CD tissues. **l**, Dot plot of IAF *IL11* expression as a function of proximity to activated macrophages. **m**, Secreted IL-11 measured from co-cultures of polarized primary human monocyte-derived macrophages, after removal of agonists, with colonic fibroblasts for 24 h. Fibroblasts only, *n* = 4; fibroblasts + macrophages, *n* = 2; fibroblasts + polarized macrophages, *n* = 3 cell lines. Mice were co-housed, and DSS treatment followed the same regimen: acute (2.0%, 7 days), chronic (2.0%, 42 days). Unless otherwise stated, statistics are from two-way analysis of variance (ANOVA) with Tukey’s multiple-comparison test on distinct biological replicates, and error bars indicate s.e.m. NS, not significant. cDC, conventional dendritic cell; IEL, intraepithelial lymphocyte; LP, lamina propria; NK, natural killer; T_reg_ cells, regulatory T cells. Scale bars, 100 μm (**a**,**f**), 10 μm (**j**). Illustrations in **g** and **m** created using BioRender. Pokatayev, V. (2025). **g**, https://BioRender.com/1ildc4h; **m**, https://BioRender.com/gp42jp3. Source Data

We modelled chronic intestinal inflammation with features of stromal-driven fibrosis using the chronic dextran sodium sulfate (DSS) regimen of three 7-day DSS cycles, each followed by a recovery phase. *Il11*^*f/f*^ and *Il11*^*f/f*^*;cre* mice were injected with tamoxifen after each DSS cycle to ablate *Il11* expression in *Il11*^*f/f*^*;cre* mice. To assess whether IL-11 promoted fibrotic collagen deposition, we performed Masson’s trichrome staining on DSS- or water-treated colon Swiss rolls and quantified the collagen-positive area as a percentage of the total tissue area. Chronic DSS exposure increased collagen deposition in *Il11*^*f/f*^ mice relative to water-treated controls, but this was reduced in DSS-treated *Il11*^*f/f*^;*cre* mice (Fig. 2a). Correspondingly, tissue hydroxyproline, a proxy for total collagen content, was elevated in DSS-treated *Il11*^*f/f*^ mice but reduced in DSS-treated *ll11*^*f/f*^*;cre* mice (Fig. 2b). Expression of profibrotic collagen genes (*Col1a1*, *Col5a1*, *Col5a2*, *Col6a1*) followed the same pattern of reduction in DSS-treated *ll11*^*f/f*^*;cre* compared with *Il11*^*f/f*^ mice (Fig. 2c), indicating that IL-11 promotes injury- and inflammation-induced fibrosis. The two genotypes exhibited shared trends with respect to weight loss and histopathological scores for tissue inflammation, indicative of a similar extent of colitis development in the absence of *Il11*; however, DSS-treated *Il11*^*f/f*^;*cre* mice exhibited reduced inflammation-driven tissue remodelling (Fig. 2d and Extended Data Fig. 3d,e). These findings extend those of previous studies that reported exacerbated colitis after acute intestinal injury of *Il11*^*−/−*^ mice and spontaneous or aggravated colitis in transgenic or recombinant IL-11-treated mice^24–26^, thereby highlighting a key role for endogenous *Il11* in promotion of fibrotic remodelling during chronic colitis.

We next used *Il11*^*mNG*^ mice to map the spatial distribution of *Il11* expression within the colon and interrogate its associated histopathological features. Following water or acute (one 7-day cycle) or chronic DSS treatment, we stained dissociated colonic cells for lineage-specific markers. mNG fluorescence was undetectable under water treatment and in non-fibroblast lineages, emerging exclusively in DSS-treated PDGFRA^+^ fibroblasts, with higher intensities observed after chronic DSS treatment (Fig. 2e). Immunofluorescence detected IL-11^mNG^ fibroblast clusters only after DSS treatment, where they localized to regions of epithelial damage as in acute intestinal injury^25,27^. Notably, IL-11^mNG^ fibroblasts accumulated in areas of pronounced collagen deposition and inflammation after repeated injury–repair cycles, modelling progressive stromal-driven tissue remodelling (Fig. 2f). These findings indicate an interplay with infiltrating immune cells that may influence IAF development or function, including collagen deposition.

To determine the relevance of *Il11*^*mNG*^ mouse fibroblasts to human IAFs, we used scRNA-seq to profile PDGFRA^+^ fibroblasts recovered after acute and chronic DSS treatment and identified ten distinct fibroblast clusters (Extended Data Fig. 3f,g). Cross-species comparison showed that mouse IAFs (mIAFs) had the strongest enrichment for human IAF and anti-TNF resistance genes^21^ (Fig. 2g and Extended Data Fig. 3h,i). Consistent with increased IL-11^mNG^ expression after chronic DSS, the relative abundance of mIAFs increased after chronic versus acute DSS (Extended Data Fig. 3j). mIAFs specifically coexpressed *Il11* and the *mNG* reporter gene, confirming the specificity of mNG for IL-11 and mIAFs (Fig. 2h). Collectively, these results identify cross-species conservation of IAFs in fibroinflammatory intestinal disease.

Given the presence of IAFs in inflamed and fibrotic tissue regions, we investigated the ligand–receptor interactions guiding their development (Methods). Among the immune cell types sending signals to IAFs, activated macrophages relayed the strongest signals, and IAFs were their top recipients (Fig. 2i). In chronic DSS-treated mouse colons, CD68 costaining confirmed close proximity between macrophages and IL-11^mNG^ fibroblasts, including direct adjacency of mNG and CD68 fluorescence (Fig. 2j). Whereas *Il11* has previously been reported to be proximal to *LysM*-expressing cells after acute intestinal injury, our data resolved their spatial association in chronic disease^25,27^. In human patients with CD and UC but not non-IBD patients, IAFs and activated macrophages cooccupied niches of active and chronic colitis, and IAF *IL11* expression was correlated with activated macrophage proximity (Fig. 2k,l). These results indicate an activated macrophage–IAF circuit in which spatial proximity may promote IAF development.

We next co-cultured primary colonic fibroblasts with monocyte-derived macrophages preconditioned for either inflammation resolution (IL-4 or TGFβ) or proinflammatory activation (IFNγ, LPS–IgG complexes or adenosine). After removal of the stimuli, only proinflammatory macrophages induced fibroblast IL-11 secretion; unstimulated or inflammation resolution macrophages had no effect (Fig. 2m). Macrophages challenged with an array of microbial- or damage-associated ligands triggered IL-11 secretion in co-cultured fibroblasts, whereas fibroblasts alone were unresponsive (Extended Data Fig. 3k). This secretion was driven by de novo transcription, as *IL11* mRNA levels increased in co-cultured fibroblasts (Extended Data Fig. 3l). Collectively, these results indicate that activated macrophages are sufficient to induce *IL11*-expressing IAFs, and spatial coassociation of these cells in vivo indicates that they may coordinate a critical inflammation-driven intercellular circuit in disease.

## TGFβ and IL-1β drive IAFs

**Extended Data Fig. 4:**
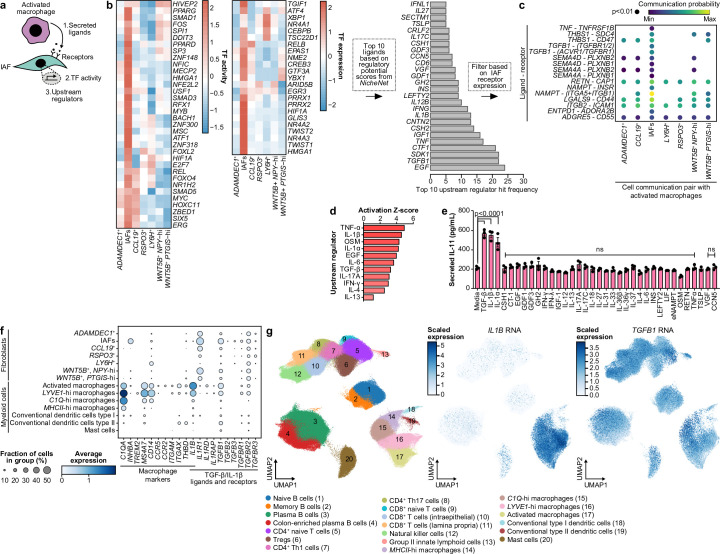
TGF-β and IL-1β drive IL-11 production. (**a**) Schematic of IAF activation inference. See Methods for more details. TF, transcription factor. (**b**) Left: heatmap of scaled cell type-averaged IAF TF activity (one-sided Wilcoxon, *P* < 0.01; mean activity difference > 0.75). Center: heatmap of pseudobulk IAF TF expression (Wilcoxon, *P* < 0.01). Right: ligand frequency (>5 among top 10 NicheNet predicted regulators). (**c**) Dot plot of ligand/receptors driving activated macrophage and fibroblast communication. (**d**) Ingenuity upstream regulator analysis of IAF genes (agonists: Z-score > 0). (**e**) Secreted IL-11 measured from primary human colonic fibroblasts stimulated with agonists from **a** (10 ng/mL, 24 h). n = 3 per condition for all except TSLP where n = 2 cell lines. One-way ANOVA with Dunnet’s multiple comparison test on distinct biological replicates and error bars are the mean ± s.e.m. ns, not significant. (**f**) Dot plot of RNA of macrophage or fibroblast markers and TGF-β and IL-1β ligands/receptors across myeloid and fibroblast subtypes. (**g**) UMAP of myeloid/lymphoid compartments alongside *IL1B* or *TGFB1* expression. Illustration in **a** was created with BioRender (https://biorender.com). Source Data

We implemented a computational approach to define the macrophage-derived signalling and gene regulatory networks driving IAF activation (Extended Data Fig. 4a–d and Methods). We inferred putative active IAF transcription factors from expression and activity scores, then predicted their converging upstream ligands using NicheNet’s ligand–receptor model^28^, restricted to expressed receptors. This approach yielded 35 candidate IAF agonists, and screening of primary colon fibroblasts with the candidate agonists demonstrated IL-11 secretion exclusively by TGFβ or by IL-1β or IL-1α (Extended Data Fig. 4e). Consistent with these results, activated macrophages showed high expression of *IL1B* as well as *TGFB1*, whereas IAFs expressed their cognate receptors (Extended Data Fig. 4f,g).

**Extended Data Fig. 5:**
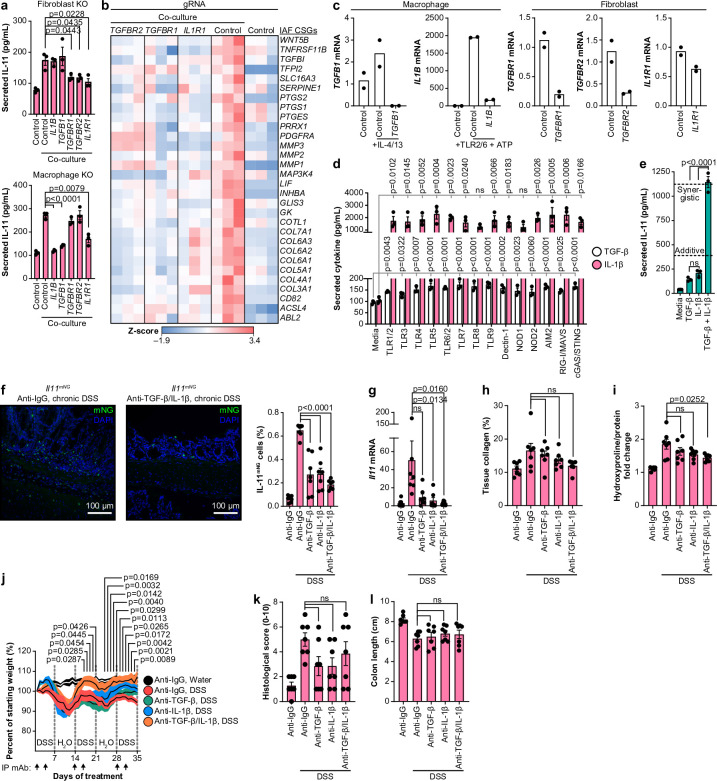
Inflammatory macrophages activate fibroblasts through both TGF-β and IL-1β. (**a**) Secreted IL-11 after co-culture of fibroblast knockouts for *TGFB1*- and *IL1B*-related ligands or receptors with TLR2/6-activated macrophages (top) or knockouts in activated macrophage knockouts with fibroblasts (bottom). n = 3 cell lines per condition. (**b**) Z-score heatmap of IAF genes in *TGFBR1/2*, *IL1R1* CRISPRko fibroblasts co-cultured with TLR2/6-activated macrophages, normalized to *HPRT*. n = 3 cell lines per condition. (**c**) qPCR of gene knockouts normalized to *HPRT*. Macrophages: IL-4/IL-13 (10 ng/mL); FSL-1 (10 ng/mL) + ATP (5 mM). Fibroblasts: media. n = 2 cell lines per condition. (**d**) Secreted TGF-β and IL-1β after co-culture of primary human macrophages and fibroblasts (24 h) (Methods). n = 3 cell lines per condition. (**e**) Secreted IL-11 from primary colonic fibroblasts stimulated with TGF-β and/or IL-1β (10 ng/mL, 24 h). Dashed lines: additive or synergistic response (Methods). One-way ANOVA with Tukey’s multiple-comparisons test. n = 3 cell lines per condition. (**f**) Left: immunofluorescence of *Il11*^*mNG*^ colons after intraperitoneal injection with IgG control or dual anti-TGF-β and anti-IL-1β antibodies (100 µL in PBS, 100 µg/mouse). Right: IL-11^mNG^ cell percentage in all DAPI-imaged cells from two pooled independent experiments. Mice (co-housed, 13-20 weeks) were treated with chronic DSS (2.0%, 35 days). n = 7 mice per condition. (**g**) qPCR quantification of *Il11* from lysates from **f** normalized to *Eef2*. Kruskal-Wallis test with Dunn’s multiple-comparison test. (**h**) Total colonic collagen percentage quantification from **f**. (**i**) Quantification of colonic hydroxyproline normalized to total protein from lysates from **f**. (**j**) Percent starting weight of mice from **f**. Filled lines represent s.e.m. Linear mixed-effects analysis with Dunnett’s multiple comparison test. (**k**) Histopathological scoring (Methods) of H&E-stained tissues from **f**. (**l**) Colon length measurements from **f**. Unless otherwise stated, statistics are by a one-way ANOVA with Dunnet’s multiple comparison test on distinct biological replicates and error bars are the mean ± s.e.m. ns, not significant. Source Data

To ascertain the contributions of TGFβ and IL-1β to IAF activation, we used knockouts of these ligands or their receptors in co-cultures of fibroblasts and TLR2- or TLR6-activated macrophages, as activated macrophages showed high expression of *TLR2*, and TLR2- or TLR6-activated macrophages were robust activators of fibroblasts (Extended Data Figs. 2e and 3k). Following TGFβ or IL-1β receptor knockout in fibroblasts, we observed impaired secretion of IL-11 and reduced expression of all measured IAF-specific genes (Extended Data Fig. 5a–c). Conversely, following *TGFB1* or *IL1B1* knockout in macrophages, IL-11 secretion was reduced in fibroblasts, demonstrating dual pathway involvement (Extended Data Fig. 5a). Notably, macrophage *IL1R1* was also required for fibroblast activation, implying autocrine feedback (Extended Data Fig. 5a). In addition to TLR2 and TLR6, diverse immune ligands induced macrophage TGFβ and IL-1β secretion, indicating that these cytokines were produced during co-culture with fibroblasts (Extended Data Fig. 5d). Stimulating fibroblasts with both cytokines led to synergistic rather than additive IL-11 production compared with individual treatments, consistent with elevated levels of IL-6 family cytokines being correlated with maladaptive tissue repair, and with a synergistic effect of this dual-cytokine stimulation regimen^29,30^ (Extended Data Fig. 5e and Methods).

To demonstrate that TGFβ and IL-1β are required to drive IL-11 production in vivo, we neutralized these cytokines with monoclonal antibodies in chronic DSS-treated *Il11*^*mNG*^ mice. Neutralization of TGFβ, IL-1β or both decreased numbers of IL-11^mNG^ fibroblasts and levels of *Il11* mRNA in the colon (Extended Data Fig. 5f,g). Cytokine blockade showed a subtle trend of alleviation of fibrosis and did not ameliorate overall inflammation (Extended Data Fig. 5h–l). The failure to rescue disease severity probably reflects disruption of broader TGFβ- and IL-1β-dependent signalling programs involved in immune homeostasis and inflammation in addition to IAFs, as receptors for both cytokines are broadly expressed in other cell types^31,32^ (Extended Data Fig. 4g). Instead, our findings identify TGFβ and IL-1β as crucial drivers of the IAF state characterized by elevated expression of *IL11* and fibroinflammatory genes.

## CRISPR screens reveal IAF determinants

**Fig. 3:**
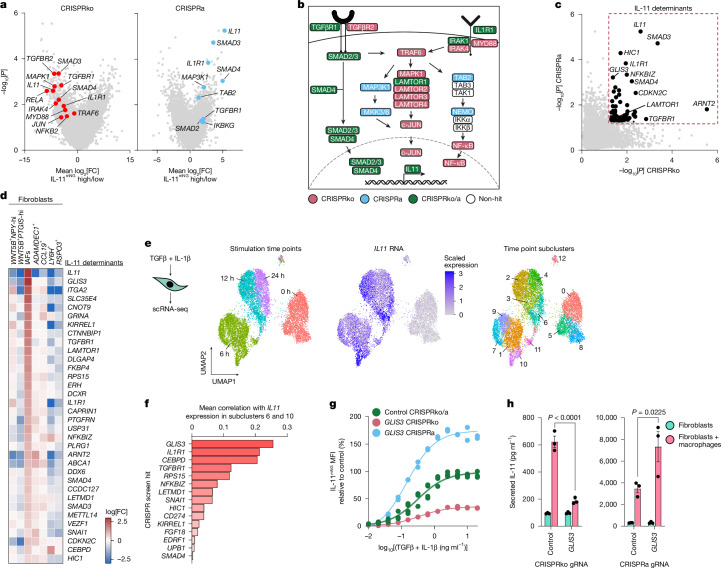
Genome-wide CRISPR screens identify novel IAF determinants. **a**, Volcano plots of enriched hits based on fold change enrichment and *P* values. One-sided hypergeometric test. Multiple comparisons adjusted by FDR. **b**, Pathway diagram of enriched hits from CRISPRko (red), CRISPRa (blue) or both screens (green) in known pathways. **c**, Scatter plot of shared hits from CRISPRko and CRISPRa screens. Statistically significant hits (*P* < 0.05) are boxed and in black, and selected hits are labelled; one-sided hypergeometric test; multiple comparisons adjusted by FDR. **d**, Heatmap depicting log fold change in expression of CRISPR screen hits across human fibroblasts. Hit selection was filtered by differential expression in IAFs (Wilcoxon test, adjusted *P* < 0.01 (two-sided); expression in more than 1% of IAFs). **e**, scRNA-seq of primary human fibroblasts stimulated with TGFβ and IL-1β (10 ng ml^−^^1^). Uniform manifold approximation and projection (UMAP) of time point clusters (left) and *IL11* expression (centre). High *IL11* expression in subclusters 6 and 10 (right). **f**, Top-ranked mean correlation values between high-*IL11*-expressing subclusters 6 and 10 from **e** and shared CRISPR screen hits from **c**; *GLIS3* showed the greatest enrichment, with *P* = 1.6 × 10^−13^ in cluster 6 and *P* = 3.9 × 10^−9^ in cluster 10. **g**, Relative percentage of IL-11^mNG^ median fluorescence intensity (MFI) in *GLIS3*-perturbed immortalized Cas9 or dCas9–VP64 fibroblasts compared with controls after TGFβ and IL-1β stimulation (24 h). Control, *n* = 4; CRISPRko/a, *n* = 2 cell lines. **h**, Secreted IL-11 from distinct biological replicates measured after co-culture of primary human colonic *GLIS3* CRISPRko (left) or *GLIS3* CRISPRa (right) fibroblasts with TLR2/6-activated monocyte-derived macrophages. Error bars indicate the s.e.m. Two-way ANOVA with Tukey’s multiple-comparison test. *n* = 3 cell lines. Panel **b** and illustration in **e** created using BioRender. Pokatayev, V. (2025). **b**, https://BioRender.com/o7ck324; **e**, https://BioRender.com/ppwff9f. Source Data

**Extended Data Fig. 6:**
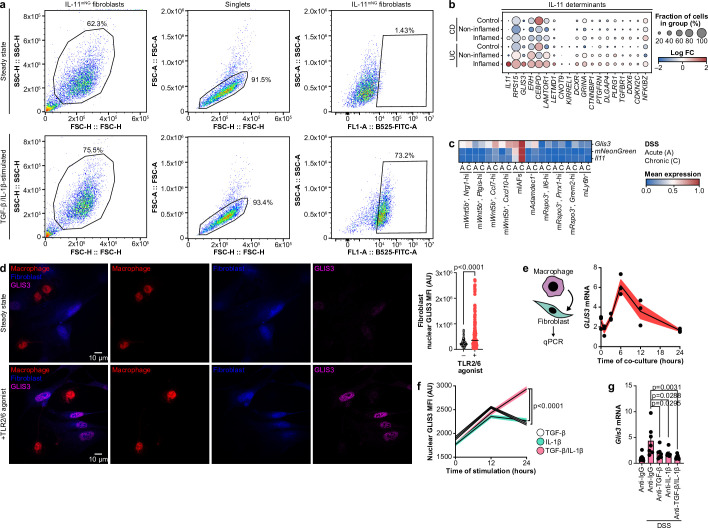
Genome-wide CRISPR screens discover novel IAF determinants. (**a**) Gating strategy to quantify IL-11^mNG^ after TGF-β and IL-1β stimulation (10 ng/mL, 24 h). (**b**) Dot plot of IL-11 determinants increased in expression during inflammation in CD and UC (Wilcoxon signed rank two-sided test, Benjamini-hochberg adjusted *P* < 0.05). (**c**) Pseudobulk scaled average expression heatmap of *Il11*, *mNeonGreen*, and *Glis3* from acute and chronic DSS-treated mice. (**d**) Left: immunofluorescence of dual-color *IL11*^*mNG*^ fibroblast-Thp-1 macrophage co-cultures with or without TLR2/6 activation. Right: nuclear GLIS3 MFI quantification. Lines represent the median. Two-tailed Mann–Whitney U test. Steady-state, n = 156; TLR2/6 stimulation, n = 186 individual cells. (**e**) qPCR measurement of *GLIS3* from primary human colonic fibroblasts co-cultured with TLR2/6-activated monocyte-derived macrophages, normalized to *HPRT*. Filled lines represent s.e.m. n = 3 cell lines. (**f**) Nuclear GLIS3 quantification in *IL11*^*mNG*^ fibroblasts with knock-in of *GLIS3*^*3XFLAG*^ stimulated with TGF-β and/or IL-1β (10 ng/mL). Filled lines represent s.e.m. One-way ANOVA with Dunnett’s multiple comparison test. n = 4 wells of median values from 6,908–12,459 fibroblasts. (**g**) qPCR measurement of *Glis3* from the sample cohort in Extended Data Fig. 5f, normalized to *Eef2*. One-way ANOVA with Dunnett’s multiple comparison test. Statistics are on distinct biological replicates and error bars are the mean ± s.e.m. ns, not significant. Illustration in **e** was created with BioRender (https://biorender.com). Source Data

Given the synergy between TGFβ and IL-1β in fibroblast activation and the need to identify fibroblast-specific targets, we investigated the signalling pathways underpinning this effect using parallel genome-wide CRISPR knockout (CRISPRko) and CRISPR activation (CRISPRa) screens anchored on *IL11* as a readout. We introduced *mNG* at the endogenous terminus of *IL11* in human fibroblasts (*IL11*^*mNG*^), resulting in an increase in fluorescence upon dual-cytokine stimulation (Extended Data Fig. 6a). We then stably expressed Cas9 or dCas9–VP64 for gene knockout or activation, respectively. After TGFβ and IL-1β stimulation, we sorted the top and bottom 15% of *IL11*^*mNG*^ expressers and sequenced guide RNAs (gRNAs) enriched in IL-11 hyperproducers or hypoproducers, respectively (Fig. 3a). Integrating the loss- and gain-of-expression screens resulted in identification of 61 shared hits, including TGFβ and IL-1β signalling components (*TGFBR1*, *TGFBR2*, *SMAD3*, *IRAK2*, *MAPK1*, *RELA*), known immunomodulatory genes (*LAMTOR*, *HIC1*, *NFKBIZ*, *ARNT2*) and *IL11* itself (Fig. 3a–c and Supplementary Data 2). Several of these genes were selectively expressed in IAFs and upregulated during inflammation, underscoring their relevance to IAF-driven disease (Fig. 3d and Extended Data Fig. 6b).

To complement these screens, we performed time-resolved scRNA-seq of fibroblasts stimulated with TGFβ and IL-1β, demonstrating *IL11* expression within 6 h of stimulation that persisted at 24 h (Fig. 3e). We observed pronounced *IL11* expression in stimulated fibroblast clusters 6 and 10, suggestive of molecular determinants within these clusters that enhanced its transcription (Fig. 3e). Correlation analysis of our bidirectional CRISPR screen hits with highly expressed genes in clusters 6 and 10 identified *GLIS3* as the top-ranked gene (*P* = 1.6 × 10^−13^ in cluster 6 and *P* = 3.9 × 10^−9^ in cluster 10) (Fig. 3f). GLIS3, a GLI-related protein that regulates pancreatic β cell development and thyroid gland function^33^, was distinctly expressed in both IAFs and mIAFs during inflammation (Fig. 3d and Extended Data Fig. 6b,c). Functional studies confirmed the role of GLIS3 in IL-11 production, as its CRISPRko impaired IL-11^mNG^ fluorescence, whereas its CRISPRa heightened it (Fig. 3g). Furthermore, during co-culture with TLR2/6-activated macrophages, IL-11 secretion was attenuated in *GLIS3* CRISPRko and elevated in CRISPRa fibroblasts (Fig. 3h).

To assess the role of GLIS3 in this macrophage–fibroblast circuit, we endogenously tagged GLIS3 (GLIS3^3xF^^lag^), and fluorescently labelled fibroblasts and macrophages with different dyes and co-cultured them with or without TLR2/6 stimulation. Activated but not resting macrophages increased fibroblast nuclear GLIS3, which formed puncta-like structures (Extended Data Fig. 6d). This increase originated from transcriptional upregulation (Extended Data Fig. 6e) and was potentiated and sustained in fibroblasts by dual TGFβ and IL-1β stimulation in an additive manner, mirroring the enhanced IL-11 production observed after dual stimulation (Extended Data Fig. 6f). Notably, in vivo neutralization of TGFβ, IL-1β or both decreased *Glis3* expression in the colon of chronic DSS-treated mice (Extended Data Fig. 6g). These results implicate nuclear GLIS3 in integration of the combinatorial effects of macrophage-derived TGFβ and IL-1β and thus in direction of *IL11* expression in IAFs.

## GLIS3 controls the IAF gene program

**Fig. 4:**
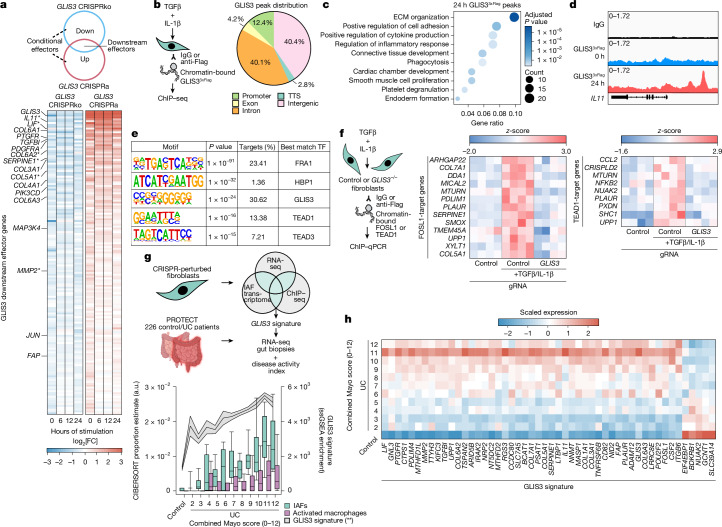
GLIS3 controls the IAF gene program. **a**, Top, Venn diagram of downregulated genes in *GLIS3* CRISPRko or upregulated in *GLIS3* CRISPRa fibroblasts after TGFβ and IL-1β stimulation (10 ng ml^−^^1^, 24 h). Significant genes with Benjamini–Hochberg-adjusted *P* < 0.05 (Wald test, two-sided) were intersected to derive effector genes. Bottom, heatmap of average log fold change in expression relative to controls. Intersecting effector genes are displayed; asterisks mark ChIP–seq peaks. *n* = 3 per condition. **b**, Schematic of ChIP–seq in *IL11*^*mNG*^ fibroblasts with *GLIS3*^*3xFlag*^ knock-in after TGFβ and IL-1β stimulation (10 ng ml^−^^1^, 24 h) (left). Pie chart depicts GLIS3^3xF^^lag^ IP peak distribution (right). **c**, Gene ontology analysis of GLIS3^3xF^^lag^ peaks at 24 versus 0 h. Benjamini–Hochberg-adjusted *P* values from hypergeometric test (one-sided). See Methods for more details. **d**, ChIP–seq tracks upstream of the first exon of *IL11* in IgG and GLIS3^3xF^^lag^ IP samples, summed across all replicate samples. **e**, Predicted transcription factor (TF) binding motifs and their associated TFs enriched in all GLIS3^3xF^^lag^ peaks. **f**, ChIP–qPCR schematic of FOSL1 and TEAD1 IP in control or *GLIS3* CRISPRko *IL11*^*mNG*^ fibroblasts after TGFβ and IL-1β stimulation (10 ng ml^−^^1^, 24 h) (left). Heatmaps depict *z*-score fold enrichment for FOSL1 (centre) or TEAD1 (right) targets across replicates. *n* = 4 cell lines per condition. **g**, Top, schematic of GLIS3 signature derivation and PROTECT cohort analysis. Bottom, CIBERSORT-estimated IAF and macrophage proportions across PROTECT samples stratified by Mayo score. Box plots represent quartiles with medians as the centre, and whiskers represent 1.5× interquartile range. Grey lines indicate mean GLIS3 single-sample gene set enrichment (ssGSEA) score ± s.e.m. **P < 0.001 (one-sided) from ordinal probit regression of Mayo scores with ssGSEA and cell proportions (*n* = 226). See Methods for more details. **h**, Heatmap of scaled average expression of refined GLIS3 signature across controls and patients with UC stratified by combined Mayo score. a.u., arbitrary units; TTS, transcription termination site. Illustrations in **b**, **f** and **g** created using BioRender. Pokatayev, V. (2025). **b**, https://BioRender.com/sk6tft5; **f**, https://BioRender.com/yr83wx3; **g**, https://BioRender.com/rw52q1c. Source Data

**Extended Data Fig. 7:**
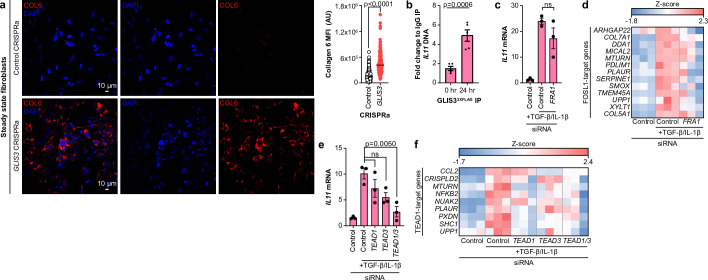
GLIS3 increases in the nucleus to regulate transcriptional control of the IAF gene program. (**a**) Left: immunofluorescence of control or *GLIS3* CRISPRa fibroblasts at steady state, stained for COL6 and DAPI (nuclei). Right: COL6 MFI quantification. Lines represent the median. Two-tailed Mann–Whitney U test. Control, n = 244; *GLIS3* CRISPRa, n = 198 individual cells. (**b**) ChIP-qPCR of *IL11* DNA in *GLIS3*^*3XFLAG*^ knock-in fibroblasts stimulated with TGF-β and IL-1β (10 ng/mL, 24 h). Unpaired Student’s t-test (two-sided). n = 5 cell lines per condition. (**c**) qPCR measurement of *IL11* in *IL11*^*mNG*^ fibroblasts treated with scrambled control or *FRA1* siRNA stimulated with TGF-β and IL-1β (10 ng/mL, 24 h), normalized to *HPRT*. Two-way ANOVA with Tukey’s multiple-comparisons test. n = 3 cell lines per condition. (**d**) Z-score heatmap for relative fold change in gene expression for FOSL1 targets after stimulation of control or *FRA1* knockdown fibroblasts with TGF-β and IL-1β (10 ng/mL, 24 h) against control non-stimulated fibroblasts, normalized to *HPRT*. n = 3 cell lines per condition. (**e**) qPCR of *IL11* in *IL11*^*mNG*^ fibroblasts treated with scrambled control or *TEAD1*, *TEAD3*, or dual *TEAD1* and *TEAD3* siRNA stimulated for TGF-β and IL-1β (10 ng/mL, 24 h), normalized to *HPRT*. Two-way ANOVA with Tukey’s multiple-comparisons test. n = 3 cell lines per condition. (**f**) Z-score heatmap for relative fold change in gene expression for TEAD1 targets after stimulation of control or *TEAD1*, *TEAD3*, or dual *TEAD1* and *TEAD3* knockdown fibroblasts with TGF-β and IL-1β (10 ng/mL, 24 h) against control non-stimulated fibroblasts, normalized to *HPRT*. n = 3 cell lines per condition. Statistics are on distinct biological replicates and error bars are the mean ± s.e.m. ns, not significant. Source Data

Having identified GLIS3 as a central regulator of *IL11* expression in IAFs, we sought to determine its broader transcriptional program through RNA-seq of *GLIS3* CRISPRko and CRISPRa fibroblasts stimulated with TGFβ and IL-1β. We identified more than 150 genes that had reduced expression in *GLIS3* CRISPRko yet enhanced expression in CRISPRa fibroblasts, indicating that they were GLIS3 effector genes (Fig. 4a). In addition to *GLIS3* and *IL11*, we observed enrichment of other IAF-specific genes implicated in fibroblast-driven pathologies, including *LIF*, which sustains fibroblast activation through positive feedback signalling^34^, and *FAP*, an IAF gene linked to intestinal strictures in CD^35^ (Fig. 4a). GLIS3 also regulated IAF gene *MMP2*, a matrix remodelling enzyme that enables trafficking of monocytes into damaged tissue, probably fuelling further macrophage–fibroblast cross-talk^18,36^, and *PTGFR* and *SERPINE1*, both of which regulate epithelial regeneration and contribute to mucosal damage during colitis^37,38^ (Fig. 4a). GLIS3 also modulated the expression of fibrotic collagens *COL6A1* and *COL6A3*, which were elevated in steady-state *GLIS3* CRISPRa compared with wild-type fibroblasts (Fig. 4a and Extended Data Fig. 7a).

To define direct GLIS3 target genes, we performed chromatin immunoprecipitation followed by sequencing (ChIP–seq) in activated *GLIS3*^*3xFlag*^ fibroblasts and identified 1,291 GLIS3-bound peaks, of which 15.2% mapped within −1 kb to +100 bp of transcription start sites and 40.4% mapped at intragenic regulatory regions (Fig. 4b). Of these peaks, 30.3% increased in enrichment after TGFβ and IL-1β stimulation, with gene ontology analysis implicating GLIS3 in the regulation of ECM organization and inflammatory response genes (Fig. 4c). These genes consisted of GLIS3 effector genes (*P* < 0.001, chi-squared test), including *IL11*, for which *GLIS3* bound upstream of the transcription start site (Fig. 4d and Extended Data Fig. 7b). To identify other transcriptional regulators that may coreside in GLIS3 peaks, we performed motif analysis and identified DNA motifs of FOSL1 (*FRA1*), a component of the AP-1 complex linked to IL-11 production in other disease models^39–43^, and TEAD1/TEAD3, downstream effectors of YAP/TAZ signalling (Fig. 4e). Notably, YAP signalling inhibition has beneficial outcomes across various organ-specific models of fibrosis^44,45^. To determine whether GLIS3 regulated the binding of FOSL1 and TEAD1 to target genes shared with GLIS3, we performed chromatin immunoprecipitation followed by quantitative PCR (ChIP–qPCR) of immunoprecipitated FOSL1 and TEAD1 from wild-type or *GLIS3* CRISPRko fibroblasts stimulated with TGFβ and IL-1β (Fig. 4f). The absence of GLIS3 impaired binding of both FOSL1 and TEAD1 to their targets, except for *IL11*, the expression of which was dependent on *GLIS3* or dual knockdown of *TEAD1*/*TEAD3* in response to TGFβ and IL-1β (Fig. 4f and Extended Data Fig. 7c–f). These data indicate GLIS3-dependent *IL11* induction in IAFs, but further regulatory contributions in other cellular contexts are plausible. Collectively, these results demonstrate that GLIS3 determines the IAF state, including *IL11* expression, and a transcriptional gene program involved in tissue remodelling and inflammation.

Having defined the GLIS3 transcriptional program in IAFs in vitro, we derived a GLIS3 signature score to quantify IAF activity in vivo. We integrated data from RNA-seq of *GLIS3*-perturbed fibroblasts, activated GLIS3-bound ChIP–seq peaks and IAF-specific transcripts to identify a core GLIS3 signature (Fig. 4g). We projected this signature on to RNA-seq profiles of colonic biopsies from more than 200 treatment-naive paediatric patients with UC from the PROTECT cohort^46^ (Fig. 4g). We observed a disease severity-dependent increase in the enrichment score of the GLIS3 signature (Fig. 4g) and further refined this signature to a subset of 50 genes, each of which was independently predictive of disease severity, including *GLIS3* and *IL11* (Fig. 4h and Methods). Deconvolution of bulk transcriptomes to infer cellular composition in the PROTECT cohort identified a direct relationship between heightened disease severity and increased frequency of IAFs and activated macrophages, factors that were both correlated with expression of the GLIS3 signature (Fig. 4g).

## GLIS3 governs intestinal fibrosis in vivo

**Fig. 5:**
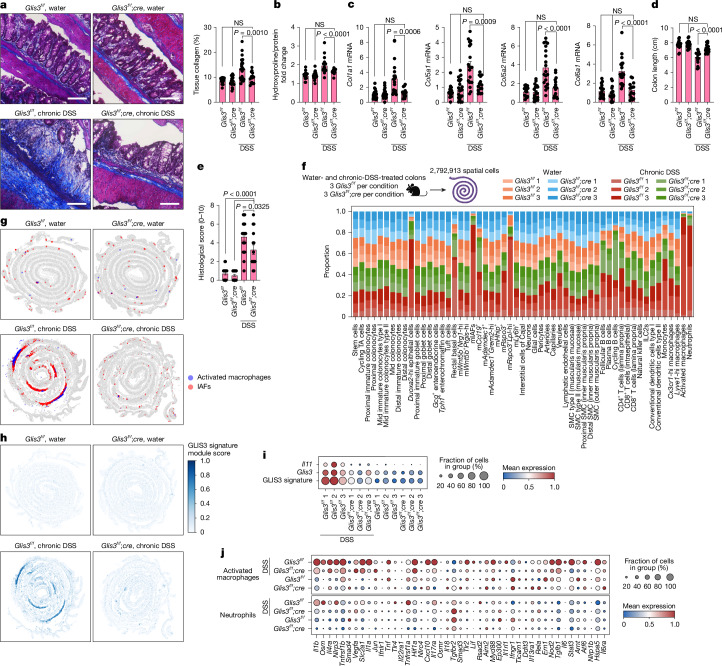
GLIS3 is required for IAF induction and aberrant collagen deposition during colitis. **a**, Masson’s trichrome-stained *Glis3*^*f/f*^ and *Glis3*^*f/f*^*;cre* mouse colons (5–18 weeks) treated with water or chronic DSS (left). Quantification of total collagen percentages across distinct biological replicates from four pooled experiments (right). *Glis3*^*f/f*^-water, *n* = 18; *Glis3*^*f/f*^*;cre*-water, *n* = 21; *Glis3*^*f/f*^-DSS, *n* = 20; *Glis3*^*f/f*^*;cre*-DSS, *n* = 15 mice. **b**, Quantification of colonic hydroxyproline normalized to total protein from tissue lysates from **a**. **c**, qPCR quantification of collagens normalized to *Eef2* from tissue lysates from **a**. **d**, Colon length measurements from **a**. **e**, Histopathological scoring (Methods) of H&E-stained tissues from **a**. **f**, Top, Xenium-based spatial profiling schematic of water- or chronic-DSS-treated *Glis3*^*f/f*^ and *Glis3*^*f/f*^*;cre* mice. Bottom, distribution of cell type proportions across water- and chronic-DSS-treated *Glis3*^*f/f*^ and *Glis3*^*f/f*^*;cre* mice. *n* = 3 mice per condition. **g**, Spatial projection of mIAFs and activated macrophages on colonic Swiss rolls of water- or chronic-DSS-treated *Glis3*^*f/f*^ and *Glis3*^*f/f*^*;cre* mice. **h**, Spatial projection of the GLIS3 signature module score on colonic Swiss rolls of water- or chronic-DSS-treated *Glis3*^*f/f*^ and *Glis3*^*f/f*^*;cre* mice. **i**, Dot plot showing expression of *Il11*, *Glis3* and the GLIS3 signature for each replicate of the Xenium spatial sequencing cohort. *n* = 3 mice per condition. **j**, Dot plot showing highlighted proinflammatory gene expression from activated macrophages and neutrophils in water- or chronic-DSS-treated *Glis3*^*f/f*^ and *Glis3*^*f/f*^*;cre* mice. *n* = 3 mice per condition. All mice were co-housed, and models of chronic DSS followed the same regimen (2.0%, 42 days). Images are representative of the sample cohort. Unless otherwise stated, statistics were obtained by two-way ANOVA with Tukey’s multiple-comparison test on distinct biological replicates, and error bars indicate the s.e.m. Scale bars, 100 μm. Illustration in **f** created using BioRender. Pokatayev, V. (2025) https://BioRender.com/olifoqi. Source Data

**Extended Data Fig. 8:**
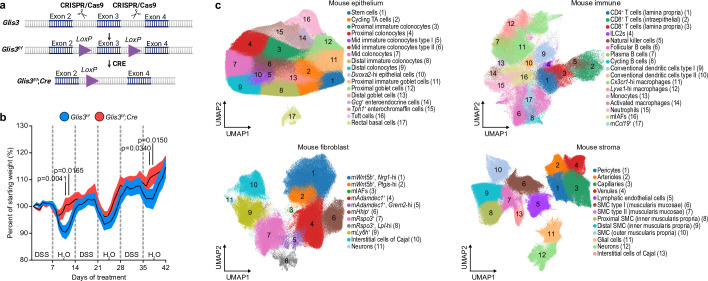
GLIS3 is required for IAF induction and aberrant collagen deposition during colitis. (**a**) Schematic of *Glis3*^*f/f*^ mice generated by flanking exon 3 with *LoxP* for Cre-mediated excision. (**b**) Percent of starting weight of *Glis3*^*f/f*^ and *Glis3*^*f/f*^;*Cre* mice from Fig. 5a. Filled lines represent s.e.m. Two-way repeated measures ANOVA. (**c**) UMAP of epithelial, immune, fibroblast, and stromal compartments in Xenium-based spatial profiling of water- and chronic DSS-treated *Glis3*^*f/f*^ and *Glis3*^*f/f*^;*Cre* mice. Illustration in **a** was created with BioRender (https://biorender.com). Source Data

To investigate the role of GLIS3 in driving intestinal pathology after chronic DSS treatment, we developed conditional *Glis3* knockout mice by flanking exon 3 with *loxP* sites (*Glis3*^*f/f*^) and crossing them to fibroblast-specific *Pdgfra*-Cre, enabling conditional knockout of *Glis3* (*Glis3*^*f/f*^*;cre*) (Extended Data Fig. 8a). Masson’s trichrome staining showed a reduction in the percentage of total colonic collagen in *Glis3*^*f/f*^*;cre* compared with *Glis3*^*f/f*^ mice treated with chronic DSS, which was mirrored by reduced tissue hydroxyproline and profibrotic collagen gene expression (Fig. 5a–c). Following chronic DSS treatment, *Glis3*^*f/f*^;*cre* mice also had lower histopathological scores for tissue inflammation, reduced weight loss and longer colon lengths compared with *Glis3*^*f/f*^ mice (Fig. 5d,e and Extended Data Fig. 8b). These results indicate a central role of *Glis3*-expressing fibroblasts in mediation of collagen deposition and driving fibroinflammatory signalling in vivo.

To further define intra- and intercellular processes by which fibroblast GLIS3 mediates fibrotic tissue remodelling, we used Xenium single-cell spatial profiling of whole-colon tissue from water- and DSS-treated *Glis3*^*f/f*^ and *Glis3*^*f/f*^;*cre* mice (Fig. 5f, Extended Data Fig. 8c). We observed no consistent differences between water-treated *Glis3*^*f/f*^ and *Glis3*^*f/f*^;*cre* mice; however, DSS-treated *Glis3*^*f/f*^;*cre* colons showed reductions in proportions of mIAFs and colocalized activated macrophages relative to those of *Glis3*^*f/f*^ (Fig. 5f,g). The GLIS3 signature, including *Il11*, showed reduced expression in *Glis3*^*f/f*^;*cre* mice (Fig. 5h,i). In addition to mIAFs and activated macrophages, we observed reductions in proportions of other immune cells in DSS-treated *Glis3*^*f/f*^;*cre* mice, particularly neutrophils (Fig. 5f). Gene expression profiling of these two myeloid subsets showed diminished levels of fibroinflammatory mediators, including *Il1b*, *Osm* and *Tnf* (Fig. 5j). Collectively, our results show that mIAFs dictate disease outcomes not only in a cell-intrinsic manner by expressing fibrotic genes but also by orchestrating intercellular interactions with proinflammatory myeloid cells in the inflamed intestine, positioning these fibroblasts as central effectors of a collapse in tissue homeostasis.

## Discussion

The relationship between tissue inflammation and homeostasis remains incompletely understood, yet it has important implications for treatment of chronic inflammatory diseases associated with fibrosis. Despite the successes of targeted biologic therapies in patients with moderate to severe IBD, only 20–30% achieve remission, and 13–46% of those patients will lose responsiveness to treatment, resulting in complications such as recurrent fibrotic strictures that necessitate surgical intervention^47,48^. As maladaptive tissue remodelling leads to fibrosis and stems from aberrant stromal cell activation, we sought to characterize the cellular and molecular architecture of IAFs and determine their roles in chronic intestinal inflammation. In both patients with CD and those with UC, IAFs and activated macrophages engaged a reciprocal cellular circuit initiated by macrophage-derived TGFβ and IL-1β at regions of tissue with active or chronic colitis. Genome-wide screens centred on this macrophage-induced fibroblast state switch identified GLIS3 as a central transcriptional regulator. Fibroblast-specific *Glis3* knockout mice were protected against intestinal inflammation, excessive collagen deposition and proinflammatory gene expression.

Our bidirectional CRISPR screens identified GLIS3 as an essential IAF regulator. Although other transcription factors including *SPI1*, *ETS1*, *TBX3*, *NR4A2* and *TWIST1* (refs. ^13,49–53^) have been reported to be involved in driving disease-associated fibroblast states, we observed greater enrichment of *GLIS3* in our functional study of IAFs. *SPI1* and *TBX3* promote myofibroblast ECM deposition, whereas *ETS1* promotes its degradation, and its loss causes increased thickening of the submucosa and muscularis propria in DSS-induced colitis^13,49,50^. In addition, *TWIST1* has been shown to be expressed in a subset of CD patient fibroblasts with high *FAP* expression, and *Col1a1*-driven *Twist1* knockout mice have less severe colitis^51,52^. Our scRNA-seq data indicated that *Fap* and *Twist1* expression mark distinct fibroblast subsets in mice—submucosal m*Grem2*^+^, m*Rspo3*^+^ fibroblasts and muscularis propria-localized m*Ly6h*^+^ fibroblasts, respectively—with *Glis3* not expressed in either of these subtypes. Notably, we observed specificity of *GLIS3* in *IL11*-expressing IAFs through mechanistic elucidation of a two-signal activation mechanism whereby TGFβ and IL-1β converge to activate GLIS3. Thus, our work positions GLIS3 as a central integrator of tissue repair processes and inflammation, inducing a disease-enriched subset of intestinal fibroblasts to express fibrosis-associated cytokines, collagen and genes associated with therapy resistance. These findings shed new light on immune–stromal cell interactions and mechanisms linking inflammation to fibrosis, raising the possibility that the IAF cell state may be conserved across tissues and diseases. Given the clinical burden of fibrotic complications in chronic inflammatory diseases and the fact that therapeutic interventions primarily act through immune suppression, the prospect of targeted antifibrotic therapy represents a promising opportunity.

## Methods

### Generating the integrated IBD scRNA-seq atlas

Previously published scRNA-seq datasets on CD and UC were used for integrative analysis^2,9,11,13^. The dataset of Smillie et al.^2^ was generated with colonic tissue biopsies obtained from 12 non-IBD controls and 18 patients with UC (from both inflamed and non-inflamed regions). Libraries were prepared for single-cell profiling by fractionating the tissues into epithelial and lamina propria fractions before downstream processing. The Kong et al.^13^ dataset was generated from 13 non-IBD controls and 46 patients with CD and consisted of biopsies obtained from inflamed regions of 17 participants and non-inflamed regions of 43 patients with disease. Libraries for single-cell profiling were prepared in a mixed manner, with some samples separated into epithelial and lamina propria fractions and some not separated. Biopsies were obtained from three segments of the gastrointestinal tract: small bowel, terminal ileum and colon. The Friedrich et al.^11^ dataset was generated by fractionation and sorting of EPCAM^−^ and CD45^−^ cells from colonic biopsies obtained from 4 non-IBD controls and 11 patients with UC, comprising 7 inflamed region biopsies and 4 non-inflamed region biopsies. The Martin et al.^9^ dataset consisted of separated lamina propria fractions of paired involved and non-involved region biopsies from 11 patients with CD. The combined dataset before downstream quality control filtering comprised 1,143,316 cells from 115 patients. Data were analysed using the scanpy implementation^54^.

### scRNA-seq analysis and cell type identification

Gene expression normalization was performed on the combined scRNA-seq dataset to account for differences in sequencing depth across cells. Unique molecular identifier (UMI) counts were normalized by the total UMI count per cell, and the total count for each cell was set to 10,000 transcripts per cell. After natural logarithm conversion and scaling of the gene expression matrix, the top 2,000 most highly variable genes were selected for a first round of dimensionality reduction. Thereafter, batch correction was performed on each individual patient sample using harmony^55^, followed by neighbourhood clustering and uniform manifold approximation and projection (UMAP) embedding of the single cells^56^. On the basis of expression of known markers of epithelial (*KRT8*, *EPCAM*), stromal (*PDGFRA*, *PECAM1*, *ACTA2*, *S100B*, *RGS5*) and immune (*CD79A*, *MZB1*, *CD3D*, *TRAC*, *C1QA*, *TPSAB*) cell populations, the clusters were then subdivided into these three main compartments for subsequent rounds of clustering and analysis. For compartment-specific analyses, gene expression normalization was performed by excluding genes with higher UMI counts (more than 5% of the total UMI count per cell) to minimize the contribution of highly expressed genes to the normalization.

In the stromal compartment dataset, only genes expressed in more than five cells were considered for further integrative analysis. Dimensionality reduction and batch correction was performed by adjusting for the following covariates: 10x Genomics Single Cell Gene Expression Solution chemistry (v.1, v.2 or v.3), patient, study and tissue site (small bowel, ileum or colon). The top 45 adjusted principal components were considered for neighbourhood clustering using the Leiden algorithm^57^ and visualized using UMAP embedding. This was followed by post hoc analysis of identified clusters to remove poor-quality cells; that is, clusters with low UMI counts or high mitochondrial gene fraction and those expressing lineage markers of non-stromal cells were removed as doublet cells. Wilcoxon rank-sum test was performed to define the markers specific to individual clusters and annotate each cluster.

Analyses for the epithelial and immune compartments followed a similar workflow to that described above, except that further rounds of iterative clustering were performed after removal of doublet and low-quality cells.

### Generation of a Xenium-based spatial transcriptomics dataset

#### Human sample collection

Sixteen patients diagnosed with UC, CD or diverticulitis who were recruited into the Prospective Registry in IBD Study at MGH (PRISM) study at Massachusetts General Hospital (MGH) participated in this study. Informed consent was obtained from all patients in accordance with the protocol approved by the institutional review board (IRB; 2004P001067). The study protocol complied with all relevant ethical regulations. Samples from patients with diverticulitis were pathologist-confirmed histologically normal. Excess tissues from clinically warranted surgical resections were collected for research purposes. IRB-approved secondary use protocol 2020P001262 allowed use of these tissues in research at the Broad Institute of MIT and Harvard.

#### Human colon sample processing

Resected human colon samples were fixed in 10% formalin overnight and stored in 70% ethanol. Tissue samples were trimmed and oriented in a tissue embedding cassette (Thomas Scientific, 230274-000B) such that all of the histological features of the colon were preserved. Paraffin embedding was done at the Koch Institute at Massachusetts Institute of Technology (MIT) histology facility to generate formalin-fixed paraffin-embedded (FFPE) blocks and stored at 4 °C. For haematoxylin and eosin (H&E) staining and pathology assessment of the tissues, FFPE tissue blocks were sectioned at 5 µm thickness on a microtome (Leica HistoCore AUTOCUTl) and placed on glass slides (VWR, Superfrost Plus). Slides were baked at 42 °C for 3 h before being stored in a desiccator overnight. Glass slides containing tissue sections were baked at 60 °C for 30 min, then H&E stained using an automatic H&E stainer at the MIT histology facility. Tissue slides were imaged with a ×10 objective using a Zeiss Axio light microscope with a Metafer slide-scanning platform. During microtome sectioning, 40 µm tissue scrolls were collected to assess tissue RNA quality with DV200 scores. These sections were placed in 1.5 ml microcentrifuge tubes and stored at −80 °C until analysis. RNA was extracted using an Qiagen RNeasy FFPE Kit (Qiagen, catalogue no. 73504), and DV200 scores were assessed with an RNA Pico chip on a 2100 Bioanalyzer (Agilent).

#### Sample preparation for Xenium profiling

Human colon FFPE blocks were sectioned at 5 µm thickness on a microtome (Leica HistoCore AUTOCUTl) and placed on Xenium glass slides (10x Genomics) following slide equilibration at room temperature for at least 30 min. Slides were baked at 42 °C for 3 h and thereafter stored in a desiccator overnight. Tissues were baked at 60 °C for 2 h, deparaffinized in xylene (MilliporeSigma, 214736-4L), ethanol rehydrated and decross-linked according to the manufacturer’s protocol (10x Genomics, demonstrated protocol CG000580 rev. C). Protocol CG000582, rev. F (10x Genomics) was used for probe hybridization, ligation and amplification for eight of the samples, whereas user guide CG000749 rev. B (10x Genomics) with further cell segmentation staining was used for the other eight human samples. A customized 480-plex gene probe panel (probe ID: HC3GPZ) was designed and used for all human samples. The probes were hybridized on to the tissue for 19 h at 50 °C. When used, the slides were incubated with cell segmentation markers for 17 h and 30 min at 4 °C. Buffers were prepared for the Xenium Analyzer instrument (software v.3.1.0.0) according to the manufacturer’s protocol for the Xenium v1 workflow (10x Genomics, user guide CG000584 rev. G). Selected regions of interest covering the full tissue of each sample were imaged, and spatially localized transcriptional data were collected.

Mouse colon tissue blocks embedded in optimal cutting temperature (OCT, Tissue-Tek) were cryosectioned at 10 µm thickness in a cryostat (Leica, CM1950) at −20 °C. Tissue sections were placed on Xenium glass slides (10x Genomics) and stored in a slide mailer at −80 °C for 4 days. Tissues were fixed in 4% paraformaldehyde (Thermo Fisher Scientific, BP531-25) and permeabilized according to the manufacturer’s protocol (10x Genomics, demonstrated protocol CG000581 rev. D). Tissue slides were further processed following the 10x Genomics user guide (CG000749 rev. B). A customized 480-plex gene probe panel (ID: JUR2CE) was used, with hybridization for 21 h at 50 °C. Tissues were stained with cell segmentation markers for 17 h and 30 min at 4 °C. Buffers for the Xenium Analyzer instrument (software v.3.2.0.1) were prepared following the Xenium v1 workflow (10x Genomics, user guide CG000584 rev. G). Slides were imaged, and transcriptional data were collected using a Xenium Analyzer instrument.

Post-Xenium tissue slides were incubated for 10 min with 10 mM sodium hydrosulfite (Sigma-Aldrich) to remove the tissue quencher (10x Genomics, demonstrated protocol CG000613 rev. B) and H&E stained using the automatic H&E stainer at the MIT Histology Facility. Glass slides were imaged on a Zeiss Axio microscope with a Metafer slide-scanning platform using a ×10 objective.

### Analysis of human spatial transcriptomics dataset

Samples were processed in two batches, with and without the cell segmentation staining kit. For tissues processed without the cell segmentation staining kit, we used a nucleus segmentation mask to restrict the counting of transcripts per cell located in its nucleus. In total, a gene expression matrix of 4,477,548 cells × 480 genes was obtained from 16 human tissues and analysed using a similar workflow to that described for the scRNA-seq dataset (see Supplementary Data 3 for the panel gene list). The analysis was implemented in Scanpy. In brief, quality control was performed to remove poor-quality cells with total RNA counts less than 10. Count normalization and log transformation were followed by regression of the effect of total RNA per cell and scaling. Principal component analysis was conducted to generate the top 50 principal components. Batch correction was then performed using the harmony package, with variables patient and batch as covariates After inspection of the elbow plot, we used the top 30 eigenvectors to construct a *k*-nearest neighbour graph with *n*_neighbors = 15. Subsequently, the Leiden clustering algorithm was applied (resolution = 0.5) to cluster the spots, and the resulting clusters were visualized with the UMAP embedding algorithm. On the basis of the expression of known markers of epithelial cells (*KRT8*, *EPCAM*), fibroblasts (*PDGFRA*, *C1S*), stromal cells (*PECAM1*, *ACTA2*, *DES*, *S100B*, *RGS5*, *CD9*, *ANO1*), lymphocyte populations (*CD79A*, *MZB1*, *CD3D*, *TRAC*), and myeloid cells (*C1QB*, *CD83*, *AIF1*, *TPSAB*, *CD36*, *FCGR3B*), the clusters were then subdivided into five main compartments for subsequent iterative rounds of clustering and analysis. In contrast to the scRNA-seq analysis workflow, we further subdivided the stromal cells into fibroblast and non-fibroblast compartments, and likewise the immune cells into lymphocytes and myeloid cells for resolution of cell type heterogeneity.

Compartment-specific analysis and clustering was implemented using a similar workflow to that described above by setting Leiden algorithm parameter resolution = 1. Clustering was followed by post hoc analysis of identified clusters to remove poor-quality cells, that is, clusters with low total RNA counts or those expressing lineage markers from other compartments. Wilcoxon rank-sum test was performed to define the markers specific to individual clusters and annotate them. Altogether, we identified 56 unique cell types across the different compartments in the spatial atlas.

### Harmonizing single-cell and spatial atlases

Analysis of the scRNA-seq IBD atlas (scIBD) revealed 51 distinct cell types, whereas 56 distinct cell types were found for the spatial IBD atlas (spIBD). Broadly, most of the cell types identified in the scIBD were also identified in the spIBD, which also recovered cell types that are known to be located deeper in the muscularis propria, that is, interstitial cells of Cajal, neurons, and smooth muscle cells located in the inner circular and outer longitudinal layer of the muscularis proporia. As the scRNA-seq was based on biopsies, these cell types were not captured. Likewise, monocytes, follicular B cells and IL6^hi^ fibroblasts were identified only in the spIBD. On the other hand, colonic plasma B cells, naive CD8 T cells, and M cells (microfold) were captured only in the scIBD.

Moreover, we observed some cell types and states that were captured in the scIBD but could not be distinguished in the spIBD. For instance, we recovered the T helper 1 (T_H_1) and T helper 17 (T_H_17) polarized states of differentiated CD4 T cells in the scIBD, but the heterogeneity between these cells could not be resolved in the spIBD; hence, they were annotated as the same cluster (CD4 T_H_1/T_H_17). Naive and memory B cells were recovered in the scIBD, but their heterogeneity could not be distinguished in spIBD. Likewise, capillaries and arteries were distinct in the scIBD but grouped into the same cluster in spIBD. *WNT5B*^+^, *NPY*^hi^ and *PTGIS*^hi^ cells were identified in the scIBD but could not be distinguished in the spIBD. Conversely, *SPP1*^+^ macrophages were identified in the spIBD, whereas *C1Q*^hi^ and *MHCII*^hi^ macrophages could not be resolved.

For the ligand–receptor analysis (described below), we focused on the common set of cell types between the two atlases and, wherever possible, used the closest cell type as a proxy when the cell types could not be identified in the spIBD.

### Cellular niche analysis on the basis of spatial transcriptomics

We evaluated the composition of cell types in the proximal neighbourhoods of each cell to identify the cellular niches (microenvironments) shared across multiple samples. For every cell in our spatial atlas, we counted the number of distinct cell types present within a radius of 30 μm, thereby generating a frequency matrix of 3,382,726 microenvironments by 56 cell types. The median number of total cells sampled within the 30-μm radius was 14 cells (range: 1–113). Next, we implemented a *k*-means clustering approach using the scikit-learn (v.0.22) package and evaluated the stability of clusters across a range of *k* values (5–35) using the silhouette score. We obtained the highest score at *k* = 19, suggesting the existence of 19 distinct niches (N0–N18) in our dataset.

### Niche and cell type enrichment analysis

Chi-squared test of independence was performed to evaluate the enrichment of cell types in a given niche. A contingency table summarizing the count distribution of each cell type across niches in the overall spatial atlas was generated, and the chi-squared statistic (*χ*^2^) was computed using observed and expected frequencies, assuming independence between variables. Degrees of freedom were calculated as follows: d.f. = (*N* − 1) × (*C* − 1), where *N* is the number of distinct niches, and *C* is the number of cell types. *P* values for cell type enrichment in each niche were obtained using standardized residuals and corrected for multiple testing using the Bonferroni method. *P* < 0.05 was considered to indicate statistical significance.

### Cell type composition analysis in single-cell and spatial atlases

To compare the compositional differences between patients with disease and healthy individuals, we used the scCODA toolbox^58^, which provides a Bayesian implementation of the Dirichlet multinomial model. For the scRNA-seq atlas, we first aggregated cell count data for each library and visualized the relative abundance of each cell type across the different conditions. Given that Martin et al.^9^ predominantly profiled immune cells, and Friedrich et al.^11^ predominantly profiled stromal cells, we restricted our analyses to the data of Kong et al.^13^ for CD and Smillie et al.^2^ for UC. Within these two studies, different tissue enriched fractions (epithelial sorted, lamina propria, immune sorted or whole tissue) were used to prepare the libraries; hence, we performed the analyses at a sample library level. scCODA was implemented on the study-specific cell count matrices by adding a pseudocount of 0.001 and considering the following covariates: patientID + enriched_fraction + disease status, using smooth muscle cells as a reference cell type because their abundance did not change between the conditions. Separate analyses were performed for patients with UC and those with CD, using the respective control participants as the reference, and a false discovery rate (FDR) < 20% was considered to indicate a significant change in abundance of the corresponding cell type. Disease-specific effect sizes (*β*) and log fold change in abundance (for cell types with credible effects only) were visualized.

For the spatial atlas, we performed the analyses at the patient level. Specifically, we first visualized the relative abundance of each niche across the different conditions, and identified niche N3 as the reference niche, as it did not change between conditions. scCODA was then implemented by adding a pseudocount of 0.001, using the non-IBD controls as reference. Niches were considered to have significantly changed in abundance if FDR < 20%. *β* values and log fold change in abundance (for niches with credible effects) were visualized.

### Differential expression analysis on scRNA-seq atlas

Pseudobulk expression for each gene was first computed for each cell type in every patient and then analysed using the decoupler and pydeseq2 packages. Differential expression was evaluated using the respective controls for UC inflamed or CD inflamed participants, including 10x chemistry as a covariate in the model. In the UC inflamed group, genes with FDR-adjusted *P* < 0.05 were considered to show statistically significant differential expression; for the CD inflamed group, this threshold was reduced to FDR-adjusted *P* < 0.1, because fewer genes showed differential regulation among these patients.

### Inference of cell communication network on the basis of spatial constraints

Intercellular cross-talk was analysed with the cellphonedb (v.5.0) package using the DEG analysis method. The scIBD was used as the reference, and separate analyses were performed for patients with UC and those with CD. First, differentially upregulated genes passing the significance threshold in inflamed tissues compared with control tissues, as described in the ‘Differential expression analysis on scRNA-seq atlas’ section, were used as the input for each cell type. As IAFs were predominantly observed in inflamed tissues, the differential expression analysis did not yield significantly upregulated genes in IAFs because of low numbers among controls. Hence, to obtain IAF upregulated genes, we performed differential expression at the pseudobulk level between IAFs and non-IAF fibroblast populations and appended the results to the input table. The threshold parameter in cellphonedb was modified to include genes with greater than 5% expression in a given cell type for scoring of interactions.

To infer spatially constrained ligand–receptor communication scores, we used the list mapping each niche to significantly enriched cell types, as described in the ‘Niche and cell type enrichment analysis’ section, as input. As some cell types identified in the scIBD could not be identified in the spIBD, as described in the ‘Harmonizing single-cell and spatial atlases’ section, we used the niche membership of the closest cell type as a proxy. Specifically, CD4 T_H_1 and T_H_17 cells in the scIBD were assumed to be members of the same niches as CD4 T_H_1/T_H_17 cells in the spatial atlas, *WNT5B*^+^*NPY*^hi^ and *WNT5B*^+^*PTGIS*^hi^ were considered to be the same as *WNT5B*^+^, capillaries and arteries were assumed to be the same as capillaries and arterioles, naive and memory B cells were assumed to be the same as naive B cells in the spatial atlas, and *C1Q*^hi^ macrophages were assumed to localize to the same niches as *LYVE1*^hi^ macrophages.

The total sum of significant ligand–receptor interaction scores were counted between a given sender–receiver pair of cell types and used to derive an adjacency matrix for network analysis. The igraph package was used to plot a directed and weighted network graph on the adjacency matrix, with edge thickness representing the sum total interaction score. Stromal compartment cells were removed from the network graph to highlight communication with IAFs and non-stromal cells.

### Histopathology annotations

H&E-stained sections of each tissue were used to manually identify structures pertaining to various anatomical and pathological regions. Using Xenium Explorer (10x Genomics), we aligned H&E images to the slide, selected the regions manually and extracted cell barcodes. We ascertained the following anatomical regions: the normal mucosa, submucosa and muscularis propria; and the following pathological regions: mucosa with predominantly active colitis, mucosa with predominantly chronic colitis, fibromuscular hyperplasia, lymphoid aggregates and granuloma. Each tissue was also qualitatively graded for stage of fibrosis, ulceration and severity of inflammation.

### Identification of putative driver ligands of IAF gene program

First, we identified transcription factors (TFs) enriched in IAFs on the basis of their expression levels. Cluster-specific enrichment analysis in the stromal compartment atlas was performed using Wilcoxon rank-sum test to identify genes enriched in IAFs. The list was then subset to the list of known TFs to yield 23 putative mediators. In parallel, we implemented DoRothEA to infer the activity of known TFs^59,60^. Then, we identified the enriched TFs in IAFs using the Wilcoxon test, considering all non-IAF stromal cells as the background. We selected TFs with a mean activity difference greater than 0.75 and *P* < 0.01, yielding a list of 34 putative TFs. The final list consisted of 55 unique TFs that putatively orchestrated the IAF program.

Next, we leveraged the ligand–target regulatory potential model available from NicheNet to identify potential ligands whose signalling converged on to the enriched TFs. Regulatory potential scores were derived by incorporating several data sources that covered ligand–receptor relationships, signal transduction and gene regulation events and then applying network propagation methods to quantify the signal flowing from a ligand through its receptors on to the signalling proteins, transcriptional regulators and, finally, target genes. For each TF in our list, we selected the top ten ligands with the strongest regulatory potential scores derived from the model. Only ligands that appeared at least five times in the top-ten list were selected for further analysis. Subsequently, we restricted the list of ligands to those whose receptors were expressed in more than 5% of IAF cells. Ultimately, this pipeline yielded 13 putative ligands, which were further expanded on the basis of ligand family membership.

### Analysis of mouse spatial transcriptomics dataset

Tissues were processed with the cell segmentation staining kit; thus, counting of transcripts was not restricted to the nucleus. In total, a dataset of 2,792,913 cells × 480 genes was generated from 12 mouse large intestines, representing three biological replicates from the following conditions: *Glis3*^*f/f*^*;cre* treated with DSS, *Glis3*^*f/f*^*;cre* treated with water, *Glis3*^*f/f*^ treated with DSS and *Glis3*^*f/f*^ treated with water. Data were analysed using a similar workflow to that described above (see Supplementary Data 3 for the panel gene list used to define cell types).

In brief, cells with total RNA counts less than 10 were removed, followed by normalization and log transformation, regression of total RNA, and scaling. The top 40 principal components were used to construct the *k*-nearest neighbour graph, and Leiden clustering was applied (resolution = 0.5), followed by visualization with UMAP. Subsequent iterative rounds of clustering were performed by grouping the clusters into four main compartments: epithelial, fibroblast, stromal and immune, including lymphocytes and myeloid cells. The following markers were broadly used: *Krt8*, *Epcam* (epithelial cells); *Pdgfra*, *Dpt* (fibroblasts); *Pecam1*, *Acta2*, *Des*, *S100b*, *Rgs5*, *Snap25*, *Ano1* (stromal cells); *Cd79a*, *Mzb1*, *Cd3d*, *Trac*, *Gata3*, *Xcl1* (lymphocytes); and *Tyrobp*, *Aif1*, *Ccr2*, *Fgr*, *C1qa*, *Cd68*, *Cd83*, *Trem2*, *Lyve1*, *Clec9a*, *Cd209a*, *Fpr1*, *Cxcr2* (myeloid cells).

Compartment-specific analysis and clustering was implemented using a similar workflow to that described above by setting Leiden algorithm parameter resolution = 1, except for the immune compartment, for which resolution = 1.5 was used. Post hoc inspection of clusters led to removal of poor-quality cells. Finally, for cell type annotation, we performed a Wilcoxon rank-sum test to define the markers specific to each cluster and inspected the spatial distribution of each cluster and the expression of cell-type-specific markers listed in Supplementary Data 3. Ultimately, we identified 55 unique cell types exhibiting distinct expression signatures and spatial distribution in the mouse large intestine.

### Mice

Animal studies complied with all relevant ethical regulations. All mice used in this study were co-housed in specific-pathogen-free facilities at MGH. For all experiments, 5- to 20-week-old male or female sex-matched mice were used. Mouse strains included C57BL/6J *Il11*^*mNG*^ mice, *Il11*^*f/f*^ mice (C57BL/6JGpt-Il11^em1Cflox^/Gpt; GemPharmatech T051922), *Glis3*^*f/f*^ mice (C57BL/6JGpt-Glis3^em1Cflox^/Gpt; GemPharmatech, T010949), R26-CreERT2 mice (B6.129-Gt(ROSA)26Sor^tm1(cre/ERT2)Tyj^/J; Jax, 008463) and PDGFRa-CRE mice (C57BL/6-Tg(Pdgfra-cre)1Clc/J, Jax, catalogue no. 013148). No statistical calculations were performed to determine sample size. Genotype-matched mice were randomly assigned to each treatment group, and no blinding was performed. Mice were housed with a 12 h dark, 12 h light cycle, at an ambient temperature of 18–24 °C and 30–70% relative humidity, and provided with food and water ad libitum. All animal procedures were conducted in accordance with protocol 2003N000158 approved by the MGH Institutional Animal Care and Use Committee, and animals were cared for according to the requirements of the National Research Council’s Guide for the Care and Use of Laboratory Animals.

### Generation of *Il11*^*mNG*^ mice

Mouse *Il11* was targeted with a gRNA verified in vitro (greater than 90% cleavage efficiency) near the stop codon of exon five (gRNA targeting sequence: TAAAGACTCGACTGTGACTC). Double-stranded DNA (dsDNA) was synthesized containing a T2A-mNeonGreen sequence flanked by approximately 400-nucleotide-long homology arms of the *Il11* gene upstream and downstream of the stop codon. A single-stranded DNA (ssDNA) sequence of this donor template was synthesized with the Guide-it Long ssDNA Production System following the manufacturer’s protocol (Takara, catalogue no. 632644). Verified gRNA, ssDNA and Cas9 with NLS (PNA Bio, CP02) complex were microinjected into zygotes by the Harvard Transgenic Mouse Core. The resulting *Il11*^*mNG*^ mice were backcrossed on the C57BL/6J background for three generations, followed by heterozygous crosses to obtain homozygous reporter mice. The licence to use mNeonGreen was acquired from Allele Biotechnology.

### Generation of *Il11*^*f/f*^;*cre* and *Glis3*^*f/f*^;*cre* mice

*Il11*^*f/f*^ mice (C57BL/6JGpt-Il11^em1Cflox^/Gpt; GemPharmatech, T051922) and *Glis3*^*f/f*^ mice (C57BL/6JGpt-Glis3^em1Cflox^/Gpt; GemPharmatech, T010949) were generated by sperm reconstitution at the UMass Chan Transgenic Animal Modeling Core on behalf of GemPharmatech. Heterozygous pups were crossed together to generate homozygous conditional knockout mice. Homozygous *Il11*^*f/f*^ mice were then bred with R26-CreERT2 mice (B6.129-Gt(ROSA)26Sor^tm1(cre/ERT2)Tyj^/J; Jax, catalogue no. 008463) to generate homozygous *Il11*^*f/f*^, hemizygous *cre* mice. *Glis3*^*f/f*^ mice were bred with hemizygous PDGFRa-CRE mice (C57BL/6-Tg(Pdgfra-cre)1Clc/J; Jax, catalogue no. 013148) to generate homozygous *Glis3*^*f/f*^, hemizygous *PDGFRa-CRE* mice.

### Acute and chronic DSS colitis

DSS experiments were performed as previously described^14^. In brief, mice were given 2.0% (weight/volume) DSS (Thermo Fisher Scientific, J14489-22) dissolved in water for 7 days for the acute model of DSS. In the chronic model, DSS administration was followed by a water recovery phase lasting for another 7 days. This cycle was repeated two more times, with mouse weights recorded daily. Upon cessation of each model (at day 7 for the acute DSS model, day 42 for *Il11* and *Glis3* mouse experiments, or day 35 for cytokine neutralization experiments), mice were euthanized, and colons were obtained for phenotyping. For *Il11* mice, chronic DSS experiments represented three pooled independent experiments with the following cohort replicates per experiment: *Il11*^*f/f*^, water (*n* = 3, 4, 11); *Il11*^*f/f*^*;cre*, water (*n* = 0, 0, 14); *Il11*^*f/f*^, DSS (*n* = 4, 3, 10); *Il11*^*f/f*^*;cre*, DSS (*n* = 3, 4, 3). For *Glis3* mice, chronic DSS experiments represented four pooled independent experiments with the following cohort replicates per experiment: *Glis3*^*f/f*^, water (*n* = 4, 4, 4, 6); *Glis3*^*f/f*^*;cre*, water (*n* = 0, 0, 0, 21); *Glis3*^*f/f*^, DSS (*n* = 4, 7, 3, 6); *Glis3*^*f/f*^*;cre*, DSS (*n* = 3, 4, 3, 5). For cytokine neutralization, chronic DSS experiments represented two pooled independent experiments with the following cohort replicates per experiment: *n* = 3, 4. Data pooling was performed by aligning animal data by timepoint (for instance, percentage weight change by day of DSS or water treatment), with all downstream analyses restricted to end point measurements.

### Tamoxifen administration

*Il11*^*f/f*^ and *Il11*^*f/f*^;*cre* mice were treated with DSS following the chronic DSS model. After cessation of each cycle of DSS administration, mice were intraperitoneally injected with tamoxifen (Sigma, T5648) dissolved in corn oil (Sigma, C8267) (200 µl of 20 mg ml^−1^). Tamoxifen was administered for 3 consecutive days, each at a different site of the mouse abdomen for each cycle of water treatment. Weights were monitored daily.

### Cytokine neutralization

*Il11*^*mNG*^ mice were treated with DSS following the chronic DSS model. At the start of each cycle of DSS administration, mice were intraperitoneally injected with monoclonal antibodies directed against IgG (BioXCell, BP0083), anti-TGFβ (BioXCell, BP0057), anti-IL-1β (Invivogen, mil1b-mab9-1T), or a combination of anti-TGFβ and anti-IL-1β. Antibodies were diluted in PBS and intraperitoneally injected at different sites of the mouse abdomen on the first and third days of each cycle of DSS administration (100 µl, 100 µg per mouse). Weights were monitored daily.

### Mouse colon cryosectioning

Water- and DSS-treated mice were euthanized with CO_2_ in accordance with the Institutional Animal Care and Use Committee protocol. Colons were removed, flushed and rinsed in PBS (Sigma, D8537-500ml), then cut longitudinally to expose the lumen. Colons were rolled from the distal to the proximal end with forceps and placed in cryo-blocks (Tissue-Tek, catalogue no. 25608-916) containing OCT (Tissue-Tek, catalogue no. 25608-930) on dry ice. Frozen colon Swiss rolls were sectioned in a Leica CM1950 cryostat at −20 °C into 10 µm slices, which were placed on to glass slides (Fisherbrand, catalogue no. 22-230-900) for imaging; and 50 µm and 100 µm slices were collected for RNA and protein extraction, respectively.

### Mouse colon staining

#### Immunofluorescence

Fresh-frozen tissue sections were fixed in 4% paraformaldehyde (Electron Microscopy Services, catalogue no. 15710-S) diluted in PBS (v/v) for 10 min at room temperature, followed by three washes in PBS. Tissues were then permeabilized in 0.2% Triton X-100 in PBS (v/v) for 10 min at room temperature, followed by three washes in PBS. Tissues were blocked with 4% bovine serum albumin (BSA; LGC Clinical Diagnostics, catalogue no. 1900-0016) in PBS (w/v) for 20 min at room temperature, then incubated with diluted primary antibodies in 4% BSA in PBS for 1 h at room temperature (1:400 for anti-mNeonGreen (Proteintech, #nfms), 1:400 for anti-CD68 (Cell Signaling Technology, 97778S)). Tissues were then washed three times in PBS and incubated with secondary antibodies in 4% BSA in PBS for 1 h at room temperature (1:500 AF-488 conjugated anti-mouse antibody (Proteintech, sms1AF488-1), 1:1,000 AF-594 conjugated anti-rabbit (Thermo Fisher Scientific, A-21207)). Tissues were washed three times in PBS, rinsed in deionized water, mounted with ProLong Diamond Antifade Mountant with DAPI (Thermo Fisher, P36962) and sealed with a cover glass (Corning, catalogue no. 2975-245) for 24 h. Images were captured on a Nikon Ti2-E inverted microscope equipped with a CSU-W1 spinning disc confocal system.

QuPath v.0.6.0 was used to quantify the percentages of IL-11^mNG^ fibroblasts in whole-colon Swiss rolls from *Il11*^*mNG*^ mice subjected to chronic DSS. Channel minimum, maximum and gamma values were set to equal measurements across the sample cohort. Cell detection was performed on the basis of DAPI-stained nuclei. IL-11^mNG^ cells were then defined by setting a single measurement classifier on objects filtered on all DAPI-stained cells. A channel filter was then set for ‘Cell: 488 nm mean’. An ‘Above Threshold’ was then set that defined IL-11^mNG+^ cells on the basis of the background signal distribution of 488 nm mean cell measurements in water-treated colon samples.

#### Masson’s trichrome staining

Staining was performed following the Masson’s Trichrome Stain Kit protocol (Vitroview, VB-3016). In brief, frozen tissue was acclimated to room temperature, fixed in 10% formalin for 30 min, and rinsed in distilled water three times. Tissues were then incubated in Bouin’s fluid for 1 h at 60 °C, washed in running tap water for 5 min, incubated with Weigert’s A+B solution mixture (1:1 mixture) for 7 min, and rinsed again with running tap water for 5 min, followed by incubation with Biebrich scarlet–acid fuschin stain for 5 min. Tissues were next rinsed in distilled water, then incubated with phosphomolybdic–phosphotungstic acid solution for a total of 10 min. Without rinsing, aniline blue solution was added for 15 min, followed by three washes in distilled water. Tissues were briefly rinsed in acetic acid, dehydrated with 90% and then 100% ethanol for 2 min each, cleared in xylene (Sigma, 214736-1L) for 5 min, three times, and finally mounted with Cytoseal 60 (EMS, catalogue no. 18007) and sealed with a cover glass. Images were captured on a Nikon Ti2-E inverted microscope for detailed magnification images or a Leica Aperio VERSA scanning system for quantification.

For quantification of colonic collagen with Masson’s trichrome-stained colon samples, FIJI imaging quantification software v.1.54p was used on images captured on the Leica Aperio VERSA scanning system. Images were increased in brightness and contrast by setMinAndMax (−12, 228). Masson’s trichrome stain deconvolution was then performed using the Colour Deconvolution 2 plugin, setting the vectors to User Values defined by Colour[1](R1: 0.412010759, G1: 0.849633569, B1: 0.329195889), Colour[2](R2:0.587269268, G2:0.67744923, B2: 0.442919122), Colour[3](R3: 0.804103811, G3: 0.584262903, B3:0.109790353). Only the blue collagen channel was used; red (muscle) and black (nuclei) signals were excluded from analyses. Collagen-positive pixel areas were identified by setting the signal threshold to 140 units to obtain the percentage of collagen-positive pixels relative to the total image area. To determine the tissue area, images were converted to 8-bit format and the signal threshold was set to 180 units; the area was then measured. The final collagen percentage was obtained by dividing the calculated collagen-positive pixel area of the tissue by the total tissue area of the image.

#### H&E staining

Staining was performed following the Hematoxylin and Eosin Stain Kit protocol (Vitroview, VB-3000). In brief, frozen tissue slides were fixed in cold 80% methanol (Sigma, 179337-1L) at 4 °C for 5 min. The slides were then placed in room temperature PBS for 10 min, followed by a rinse in running deionized water for 2 min. Next, slides were placed in Mayer’s Hematoxylin Solution for 5 min and then rinsed in running tap water for 5 min; placed in 95% ethanol for 1 min and then eosin solution for 1 min 30 s; and dehydrated by being placed in two changes of 95% ethanol for 2 min each, and two changes of 100% ethanol for 2 min each. The tissues were cleared in xylene (Sigma, 214736-1L) for 5 min, three times, and finally mounted with Cytoseal 60 (EMS, catalogue no. 18007) and sealed with a cover glass. Images were captured on a Leica Aperio VERSA scanning system.

### Histopathological quantification of tissue inflammation

Blinded histopathological assessment of H&E-stained tissues was carried out by a trained pathologist (A.R.S.). H&E-stained slides were graded by a standardized score of tissue injury and inflammation as described previously^61^. The total histological score ranged from 0 to 10 and was derived from the sum of four scoring criteria: mucosal ulceration, epithelial hyperplasia, lamina propria mononuclear infiltration and lamina propria neutrophil infiltration. Mucosal ulceration scoring criteria were either normal (0), surface epithelial inflammation (1), erosions (2) or ulcerations (3). Epithelial hyperplasia scoring criteria were either normal (0), mild (1), moderate (2) or pseudopolyps (3). Lamina propria mononuclear infiltration scoring criteria were either normal (0), slightly increased (1) or markedly increased (2). Lamina propria neutrophil infiltration were either normal (0), slightly increased (1) or markedly increased (2).

### Hydroxyproline determination

First, 100-µm slices of OCT-embedded frozen colonic tissues were obtained by cryosectioning and flash frozen in liquid nitrogen. Tissues were crushed using manual dissociation on dry ice and incubated in Pierce RIPA buffer (Thermo Fisher, catalogue no. 89900) for 20 min. Following a freeze–thaw cycle, hydroxyproline extraction was performed following the manufacturer’s protocol with a few modifications (Sigma, MAK463-1KT). Fifty microlitres of lysates were incubated with 50 µl of 10 N NaOH for 2 h at 100 °C under constant agitation. Samples were allowed to cool to room temperature, then neutralized by addition of 50 µl of 10 N HCl, diluted 1:1 with 150 µl of water, and centrifuged for 5 min at 14,000*g* at room temperature to remove any particulates. Lysates were transferred to new tubes. A hydroxyproline standard (range: 0–50 µg ml^−1^) was then prepared, and 20 µl of each standard and tissue lysate were transferred to individual wells on a 96-well plate (Corning, catalogue no. 29444-018). A reaction mixture containing 8 µl of reagent A and 90 µl of oxidation buffer was prepared for all samples, and 90 µl of this mixture was added to each well, followed by 10 min of incubation at room temperature. Then, 90 µl of reagent B was added to all wells, and the contents of the wells were mixed by pipetting up and down until turbidity dissipated. The plate was incubated for 90 min at 37 °C, after which the optical density of each well was measured at 560 nm on a BioTek Synergy H4 Hybrid Multi-Mode Microplate Reader. To quantify the amount of hydroxyproline, the optical density values of the standards were plotted against their concentrations. Each sample’s optical density measurement was then divided by the slope of the standard curve to yield a hydroxyproline concentration in micrograms per millilitre. Values were normalized to protein concentrations determined by measurements from a bicinchoninic acid (BCA) assay.

### Protein concentration determination

Total protein concentrations were determined using a Pierce BCA protein assay kit (Thermo Fisher, catalogue no. 23225) on prepared tissue lysates with a few modifications. A standard curve with Pierce BSA standard (Thermo Fisher, catalogue no. 23209) was prepared (range: 0–10 µg µl^−1^) using individual wells of a 96-well plate, and 1 µl of sample was added to each well. A reaction mixture was then prepared by adding 50 parts of BCA reagent A with 1 part of BCA reagent B, and 200 µl of BCA reaction mixture was added to each well of the 96-well plate, followed by incubation of the plate for 30 min at 37 °C. The optical density of each well was measured at 562 nm on a BioTek Synergy H4 Hybrid Multi-Mode Microplate Reader. Each sample’s optical density measurement was then divided by slope of the standard curve to yield a protein concentration in micrograms per microlitre.

### Mouse colon dissociation

Mouse colons were dissociated following a modified protocol^62^. Following acute or chronic DSS treatment, mice were euthanized, and colons were placed in ice-cold HBSS (Thermo Fisher, catalogue no. 14170112). Colons were cut longitudinally and then cut into small pieces. The tissue was then placed in 15 ml of epithelial cell solution (10 mM HEPES (Thermo Fisher, catalogue no. 15630080), 0.5 M EDTA (Thermo Fisher, catalogue no. AM9260G), 100 U ml^−1^ penicillin–streptomycin, 2% fetal bovine serum (FBS), 100 μg ml^−1^ DNase I (Roche, catalogue no. 10104159001) in HBSS). Samples were incubated at 37 °C for 15 min and manually shaken every 5 min. After incubation, samples were placed on ice for 10 min. Tissue pieces were then transferred to new tubes with HBSS and vigorously shaken for 40 s. The supernatant was collected by filtering through a 100-μm cell strainer (Corning, catalogue no. 352360) into a new tube, and pieces of tissue were placed in the original tube. Shaking and filtering were performed a further two times to dissociate and deplete this epithelial fraction. The remaining tissue was placed in HBSS and washed with gentle shaking three times, for 30 s each time. With a razor blade, the tissue was cut into smaller pieces and transferred to a new tube containing 10 ml of lamina propria solution (100 U ml^−1^ penicillin–streptomycin, 100 μg ml^−1^ Liberase (Sigma, catalogue no. 5401127001), 100 μg ml^−1^ DNase I in RPMI). Tissues were incubated 37 °C for 20 min. Manual pipetting with a 5-ml pipette was used to break apart the tissue. The tissue was then incubated at 37 °C for a further 10 min, followed by manual pipetting with a 1-ml pipette to break apart the tissue further until dissociation. Samples were transferred to ice, and 1 ml of FBS and 80 μl of EDTA were added to stop the digestion reaction. Tissue samples were pipetted again with a 1-ml pipette and filtered with a 40-μm cell strainer (Corning, catalogue no. 352340). HBSS was added, and cells were spun down at 400*g* for 10 min.

### Cell surface staining of colonic cells

Isolated colonic cells were washed in PBS and resuspended in PBS with 2% FBS. Cells were then Fc blocked (BioLegend, catalogue no. 101320) on ice for 10 min at 1 μg per 100 ml buffer. Without removal of the Fc block, a master-mix of fluorophore-conjugated antibodies diluted 1:200 each in PBS with 2% FBS was added to the cells for 30 min on ice (APC anti-mouse CD45 (BioLegend, catalogue no. 157605), PE/Dazzle 594 anti-mouse TER-119 (BioLegend, catalogue no. 116243), Brilliant Violet 421 anti-mouse PDGFRA (BioLegend, catalogue no. 135923), PE anti-mouse PECAM1 (BioLegend, catalogue no. 102407), Brilliant Violet 711 anti-mouse EPCAM1 (BioLegend, catalogue no. 118233) and LIVE/DEAD Fixable Near IR (780) viability stain (Thermo Fisher, L34992)). Cells were spun at 500*g* for 3 min and washed with PBS. This spin and wash was repeated; then, cells were resuspended in live cell sorting buffer (PBS with 5% FBS, 25 mM HEPES, 1 mM EDTA) and sorted on a Sony SH800 cell sorter. Single-antibody stains for each fluorescent channel using compensation beads (Beckman Coulter, B22804) were used to set up compensation.

### Cell culture

Primary colon fibroblasts (CRL-1459), and immortalized fibroblasts (CRL-4001) were obtained from the American Type Culture Collection. Lenti-X 293T cells for lentivirus production were obtained from Takara (catalogue no. 632180). Primary-monocyte-derived macrophages were from isolated from healthy donor blood (unpurified buffy coats, 25–50 ml; ages within 18–65 years, unknown distribution of male and female) were collected at Research Blood Components, LCC, MA, USA, after a signed consent form had been obtained. Standard testing for blood-borne pathogens was performed. THP-1 Cas9-expressing cells were a gift from the Genomics Platform of the Broad Institute of MIT and Harvard. Commercially purchased cell lines were authenticated by the American Type Culture Collection with short tandem repeat identification. No authentication of Lenti-X 293T cells was provided by Takara, nor were donor or donated cells authenticated. All cell lines tested negative for mycoplasma except donor-derived cells, which were not tested.

Fibroblasts and Lenti-X 293T cells were cultured in Dulbecco’s modified Eagle’s medium (Thermo Fisher Scientific, catalogue no. 10569044) supplemented with 10% (v/v) heat-inactivated FBS (MilliporeSigma, F2442), 1× penicillin–streptomycin (Corning, 30-002-CI). Myeloid cells were cultured in RPMI-1640 medium (Gibco, catalogue no. 22400-089) supplemented with 10% FBS and 1× penicillin–streptomycin. All cells were cultured at 37 °C, with atmospheric O_2_ and 5% CO_2_.

### Human monocyte purification and macrophage differentiation

Human monocytes from unpurified buffy coats were isolated with RosetteSep Human Monocyte Enrichment Cocktail (STEMCELL, catalogue no. 15028) following the manufacturer’s protocol, then enriched with SepMate tubes and Ficoll-mediated separation. Isolated cells were washed with PBS before being plated in RPMI-1640 containing recombinant M-CSF (PeproTech, catalogue no. 300-25) for 7 days on non-treated petri dish plates (Falcon, catalogue no. 351029). For polarization to different macrophage subsets, on day 6 following monocyte isolation, attached macrophages were washed with PBS and incubated with macrophage-polarizing ligands for 24 h (see Supplementary Data 4 for concentrations and catalogue numbers). For differentiation of THP-1 Cas9 monocytes into adherent macrophages, cells were stimulated with 300 ng ml^−1^ phorbol myristate acetate (Invivogen, tlrl-pma) for 3 h at 37 °C on non-treated petri dishes. Cells were then washed in PBS and reseeded on tissue-culture-treated plates for 2 days in RPMI-1640.

### Cell stimulation

Fibroblasts were first seeded on a multiwell tissue-culture plate. Following adherence at 75–90% confluency overnight, cells were washed with PBS, and the appropriate ligand diluted in complete DMEM was added for the indicated time (Supplementary Data 4). Following stimulation, plates were spun down, and media supernatant was collected for analysis, or cells were washed with PBS and collected into TRIzol reagent (Thermo Fisher Scientific, catalogue no. 15596026) for RNA extraction.

### Calculation of additive or synergistic IL-11 production

The following mathematical formulation published by Sanford et al.^63^ was used to determine whether IL-11 was produced additively or synergistically with dual TGFβ and IL-1β stimulation. The amount of IL-11 produced in response to single cytokine stimulation or at baseline was set as the following variables.

$$\begin{array}{l}\mathrm{Expression\; of\; IL}\mathrm{-}\mathrm{11\; at\; baseline}={X}_{\mathrm{baseline}}\\ \mathrm{Expression\; of\; IL}\mathrm{-}{\rm{11\; after\; TGF}}\beta ={X}_{\mathrm{baseline}}+\Delta {\rm{TGF}}\beta \\ {\rm{Expression\; of\; IL}}\mathrm{-}{\rm{11\; after\; IL}}\mathrm{-}1\beta ={X}_{\mathrm{baseline}}+\Delta {\rm{IL}}\mathrm{-}1\beta \end{array}$$

$$\begin{array}{l}{\rm{I}}{\rm{f}}\,{\rm{e}}{\rm{x}}{\rm{p}}{\rm{r}}{\rm{e}}{\rm{s}}{\rm{s}}{\rm{i}}{\rm{o}}{\rm{n}}\,{\rm{o}}{\rm{f}}\,{\rm{I}}{\rm{L}}\mathrm{-}11\,{\rm{a}}{\rm{f}}{\rm{t}}{\rm{e}}{\rm{r}}\,{\rm{T}}{\rm{G}}{\rm{F}}{\rm{\beta }}\,{\rm{a}}{\rm{n}}{\rm{d}}\,{\rm{I}}{\rm{L}}\mathrm{-}1{\rm{\beta }}\,{\rm{i}}{\rm{s}}\,{\rm{a}}{\rm{d}}{\rm{d}}{\rm{i}}{\rm{t}}{\rm{i}}{\rm{v}}{\rm{e}}\\ \,\,\,=\,{{\rm{X}}}_{{\rm{b}}{\rm{a}}{\rm{s}}{\rm{e}}{\rm{l}}{\rm{i}}{\rm{n}}{\rm{e}}}+\Delta {\rm{T}}{\rm{G}}{\rm{F}}{\rm{\beta }}+\Delta {\rm{I}}{\rm{L}}\mathrm{-}1{\rm{\beta }}\\ \,\,\,=\,388.8\end{array}$$

$$\begin{array}{c}\mathrm{If\; expression\; of\; IL}\mathrm{-}{\rm{11\; after\; TGF\beta \; and\; IL}}\mathrm{-}1\beta \,\mathrm{is\; synergistic}\\ =\,{{\rm{X}}}_{\mathrm{baseline}}\times \mathrm{fold\; change}\,\Delta \mathrm{TGF}{\rm{\beta }}\times \mathrm{fold\; change}\,\Delta \mathrm{IL}\mathrm{-}1{\rm{\beta }}\\ =\,{{\rm{X}}}_{\mathrm{baseline}}\times (({{\rm{X}}}_{\mathrm{baseline}}+\Delta \mathrm{TGF\beta })/{{\rm{X}}}_{\mathrm{baseline}})\times (({{\rm{X}}}_{\mathrm{baseline}}+\Delta \mathrm{IL}\mathrm{-}1{\rm{\beta }})/{{\rm{X}}}_{\mathrm{baseline}})\\ =\,{{\rm{X}}}_{\mathrm{baseline}}+\Delta \mathrm{TGF\beta }+\Delta \mathrm{IL}\mathrm{-}1{\rm{\beta }}+(\Delta \mathrm{TGF\beta }\times \Delta \mathrm{IL}\mathrm{-}1{\rm{\beta }})/{{\rm{X}}}_{\mathrm{baseline}}\\ =\,1123\end{array}$$

### RNA isolation and RT–qPCR

#### In vitro cell cultures

RNA from in vitro cell cultures was isolated as previously described^64^. RNA was extracted from cells in TRIzol reagent following the manufacturer’s protocol with chloroform and isopropanol precipitation, followed by a wash in 75% ethanol (Thermo Fisher Scientific).

#### Tissue samples

RNA from in vivo tissue samples were isolated with an Aurum Total RNA Mini Kit (Bio-Rad, catalogue no. 7326820) following the manufacturer’s instructions. Then, 50-µm slices of OCT-embedded frozen colonic tissues were obtained by cryosectioning and flash frozen in liquid nitrogen. Tissues were crushed using manual dissociation on dry ice and then lysed in 700 μl of lysis solution, with pipetting up and down to lyse the tissue. The lysate was centrifuged for 3 min at room temperature at 14,000*g* and then transferred to a new tube; then, 700 μl of 60% ethanol was added to the lysate, and the mixture was transferred to an RNA binding column. The column was centrifuged for 60 s at room temperature at 14,000*g*, after which the filtrate was discarded. Next, 700 μl of low stringency wash solution was added to the column, and samples were centrifuged for 30 s at room temperature at 14,000*g* to remove the solution. An 80-μl mixture of DNase I was added to the column and allowed to digest at room temperature for 25 min; after this, 700 μl of high stringency wash solution was added to the column, and samples were centrifuged for 30 s at room temperature at 14,000*g* to remove the solution. Low stringency wash solution (700 μl) was again added to the column, and samples were centrifuged for 30 s at room temperature at 14,000*g* to remove the solution. The column was then centrifuged for 2 min at room temperature at 14,000*g* to remove any remaining liquids. RNA was eluted by addition of 40 μl RNA elution solution, followed by centrifugation for 2 min at room temperature at 14,000*g* to collect the RNA.

Equal amounts of RNA were used to synthesize cDNA with an iScript cDNA synthesis kit (Bio-Rad Laboratories, catalogue no. 1708891). iTaq Universal SYBR Green Supermix (Bio-Rad Laboratories 1725124) was used for quantitative PCR with reverse transcription (RT–qPCR) on a C1000 Touch Thermal Cycler (Bio-Rad Laboratories). Relative gene expression was calculated using the 2^−ΔΔCT^ method^65^, with *HPRT* as the reference housekeeping gene for human samples and *Eef2* for mouse samples. See Supplementary Data 5 for primer sequences. Heatmaps of gene expression *z*-scores were generated in Microsoft Excel v.16.

### Secreted protein quantification

Secreted cytokines were measured by spinning down cells and debris of cell culture media. IL-11, IL-1β and TGFβ were measured with a cytometric bead array assay (BD Biosciences, catalogue nos 560228, 558279, 560429) following the manufacturer’s protocol. Samples were measured for median fluorescence intensity of the cytokine of interest using a CytoFLEX S cytometer (Beckman Coulter). Median fluorescence intensity readings for each cytokine were plotted against a standard curve to obtain final cytokine concentrations. Data were analysed using FlowJo v.10.8 (BD Life Sciences)^66^.

### Generation of CRISPR knock-in fibroblasts

#### *IL11*^*mNG*^

Human *IL11* was targeted with a gRNA verified in vitro (greater than 90% cleavage efficiency) near the stop codon of exon 5 (gRNA targeting sequence: TGAAGACTCGGCTGTGACCC). dsDNA was synthesized containing a T2A-mNeonGreen sequence flanked by approximately 400-nucleotide-long homology arms of the *IL11* gene upstream and downstream of the stop codon. ssDNA was then synthesized with the Guide-it Long ssDNA Production System following the manufacturer’s protocol. Cells were electroporated with the ssDNA, along with Cas9 with nuclear localization signal (PNA Bio, CP02) and *IL11*-targeting gRNA using a Lonza 4D-Nucleofector X Unit kit (Lonza, AAF-1003X) with SE Cell Line 4D-Nucleofector X Kit S (Lonza, V4XC-1032). Cells were expanded, and single-cell clones were sorted for mNeonGreen expression following fibroblast activation. Positive clones were then stably infected with lentivirus encoding Cas9 (pXPR_101) or dCas9–VP64 (pXPR_109) (plasmids were obtained from the Genetic Perturbation Platform of Broad Institute of MIT and Harvard), and activity for CRISPRko or CRISPRa was verified with gRNAs. Cells were passaged under blasticidin S HCl (Thermo Fisher Scientific, A1113903) to maintain Cas9 expression.

#### GLIS3^3xFlag^

For 3xFlag-tagged GLIS3 fibroblasts, a 3xFlag sequence flanked by short homology arms to the *GLIS3* insertion site was synthesized and electroporated (as described above for *IL11*^*mNG*^) into *IL11*^*mNG*^ Cas9 fibroblasts, along with a gRNA targeting *GLIS3* (gRNA targeting sequence: CCAAGAGAGCTTTTAGCCTT). Cells were single-cell cloned, and 3xFlag knock-in cells were selected and verified for Flag expression with an anti-Flag antibody (MilliporeSigma, F1804-200UG) in an image-based assay (‘Immunofluorescence staining’).

### CRISPRko, CRISPRa and siRNA cell line generation

#### Lentiviral gRNA cloning

Oligos targeting a gene of interest were designed on the basis of CRISPick of the Genetic Perturbation Platform of the Broad Institute of MIT and Harvard. See Supplementary Data 5 for sequences. Oligos were annealed and phosphorylated using T4 ligation buffer (New England Biolabs, M0202L) and T4 PNK (New England Biolabs, M0201L). Esp3i-digested (Thermo Fisher Scientific, FD0454) pXPR_003 or pXPR_502 for CRISPRko or CRISPRa, respectively, were then ligated with annealed oligos using quick ligase (New England Biolabs, M2200L). Plasmids were transformed into One Shot Stbl3 chemically competent *Escherichia coli* cells (Thermo Fisher Scientific, C737303), single colonies were selected, and DNA was extracted using a spin miniprep kit (Qiagen, catalogue no. 27106). Correct integration of gRNA sequences was verified by Sanger sequencing.

#### Lentivirus production

Lenti-X 293 cells were seeded and transfected with a combination of psPAX2 (Addgene, catalogue no. 12260), VSV-G (Addgene catalogue no. 8454), and CRISPRko or CRISPRa vectors in Opti-MEM I reduced serum media (Thermo Fisher catalogue no. 31985088) and complexed to Lipofectamine 2000 (Thermo Fisher Scientific, catalogue no. 11668019). After 16 h of incubation of cells with the multiplasmid complex, media were replenished, and cells were allowed to grow for 48 h. Media were then collected and spun to remove debris, and the supernatant containing lentivirus was frozen at −80 °C. *IL11*^*mNG*^ Cas9 or dCas9–VP64 fibroblasts were stably infected with lentivirus-containing gRNAs targeting the gene of interest. Cells were then selected using puromycin (Thermo Fisher Scientific, A11138-03) and blasticidin.

#### Ribonucleoprotein knockout

Knockout cells were generated with gRNA–Cas9 electroporation, following the recommendations of the manufacturer (Integrated DNA Technologies). In brief, a synthesized gRNA for the gene of interest was obtained through Integrated DNA Technologies and resuspended in water. The ribonucleoprotein complex was formed by incubating 120 pmol gRNA with PBS and 104 pmol of Alt-R Cas9 enzyme (Integrated DNA Technologies, catalogue no. 1081058) at room temperature for 20 min. Cells were electroporated with the resultant ribonucleoprotein complex using a Lonza 4D-Nucleofector X Unit kit with SE Cell Line 4D-Nucleofector X Kit S.

#### Small interfering RNA

Predesigned small interfering RNA (siRNA) oligomers were purchased from Sigma-Aldrich and resuspended in nuclease-free water at 20 μM (scrambled control (Sigma, SIC001), *FRA1* (Sigma, SASI_Hs01_00191186), *TEAD1* (Sigma, SASI_Hs01_00031302), *TEAD3* (Sigma, SASI_Hs01_00090158)). Seeded fibroblasts were transfected with 150 pmol siRNA complexed with Lipofectamine RNAiMAX (Thermo Fisher, catalogue no. 13778030) in Opti-MEM media (Thermo Fisher, catalogue no. 31985062). Twenty-four hours later, cells were reseeded into 12-well plates and plated in triplicate knockdown per condition; another 24 h later, cells were washed with PBS and replenished with fresh media with or without addition of 10 ng ml^−1^ of human TGFβ and IL-1β for 24 h. Cells were washed in PBS and resuspended in TRIzol reagent for RNA isolation.

### Fibroblast and macrophage co-culture

On day 6 after M-CSF treatment of primary human monocytes, differentiated macrophages were polarized with agonists for 24 h (for concentrations, see Supplementary Data 4). Following removal of agonist and several washes in PBS, fibroblasts were seeded with the macrophages for 24 h. For experiments using THP-1 macrophages, following 2 days of rest after phorbol myristate acetate stimulation, fibroblasts were seeded on to the differentiated THP-1 macrophages, and agonist was added to the media.

For qPCR-based measurements of fibroblasts in co-culture, fibroblasts were seeded at the bottom of a 24-well plate, with macrophages separated through a transwell insert (Corning, catalogue no. 3462). Following co-culture, macrophages were removed, and RNA was isolated from fibroblasts as described above.

### Immunofluorescence staining

#### Monoculture of fibroblasts

Cells were seeded on a coverglass (Celltreat, catalogue no. 229173) in a 12-well tissue-culture dish. After completion of the experiment, they were fixed in 2% paraformaldehyde, followed by three washes in PBS for 5 min each time. Cells were then permeabilized with 0.2% Triton X-100, washed in PBS for 5 min, blocked with 4% BSA–PBS and incubated with 1:500 Flag antibody (MilliporeSigma, F1804-200UG) or 1:500 collagen 6 antibody (Thermo Fisher Scientific, PA5-106556) in 4% BSA–PBS for 1 h at room temperature. Cells were then washed again with PBS and incubated with a 1:1,000 dilution of AF-647-conjugated anti-mouse antibody (Thermo Fisher Scientific, A-21235) or a 1:1,000 dilution of AF-594 anti-rabbit antibody (Thermo Fisher Scientific, A-21207) in 4% BSA–PBS for 1 h. Finally, cells were washed three times in PBS, rinsed in distilled water, and mounted with ProLong Diamond Antifade Mountant with DAPI on to a glass slide for 24 h. Images were captured on a Nikon Ti2-E inverted microscope equipped with a CSU-W1 spinning disc confocal system.

#### Co-cultures of fibroblasts and macrophages

Fibroblasts and THP-1 macrophages were prestained with fluorescent labelling dyes before co-culture. In brief, differentiated macrophages were labelled with CellTracker Orange CMRA Dye at 10 µM (Thermo Fisher Scientific, C34551) for 30 min at 37 °C in serum-free RPMI-1640 medium. Cells were then washed in PBS and seeded on to a coverglass in a 12-well dish in complete RPMI-1640 overnight. The following day, adherent fibroblasts were labelled with CellTracker Blue CMAC Dye at 10 µM (Thermo Fisher Scientific, C2110) for 30 min at 37 °C in serum-free RPMI-1640 medium. Cells were then washed, trypsinized and seeded on to the labelled macrophages along with macrophage-activating ligand FSL-1 (1 ng ml^−1^; Invivogen, tlrl-fsl) overnight. Immunofluorescent staining was performed as above (‘Monoculture of fibroblasts’). Images were captured on a Nikon Ti2-E inverted microscope equipped with a CSU-W1 spinning disc confocal. Cells were then washed with PBS and imaged again.

FIJI imaging quantification software (v.1.54p) was used to quantify the fluorescence intensity of AF-647-labelled nuclear *GLIS3*^3xF^^lag^ or AF-594-labelled collagen 6. The median fluorescence intensity of AF-647-labelled nuclear *GLIS3*^3xF^^lag^ was quantified in each individual fibroblast nucleus.

### Statistics and reproducibility

Statistical analyses were performed using GraphPad Prism v.10. One-way analysis of variance (ANOVA) was used for comparisons among three or more groups. Two-way ANOVA was used to compare factorial groups. For Tukey’s, Dunnet’s and Dunn’s multiple-comparison tests, the family-wise error rate was set to a level of 0.05 (95% confidence interval). Multiplicity-adjusted *P* values less than 0.05 were considered to indicate statistical significance. All experiments were repeated at least twice as successful, independent experiments.

### CRISPR screens

Lentivirus stocks of the Brunello CRISPRko and Calabrese CRISPRa libraries were obtained from the Genomics Platform of the Broad Institute of MIT and Harvard. Viral titres were calculated using a puromycin selection assay in which a target multiplicity of infection of 0.3 was selected so that each individual fibroblast could be targeted with one gRNA. *IL11*^*mNG*^ Cas9 or *IL11*^*mNG*^ Cas9–VP64 fibroblasts were infected with the determined titres of the CRISPRko or CRISPRa libraries, respectively, at 500× representation of each library for 24 h in DMEM supplemented with 10 µg ml^−1^ polybrene (MilliporeSigma, TR-1003-G). Fibroblasts were washed in PBS the following day, then incubated with 1 μg ml^−1^ puromycin and 3 μg ml^−1^ blasticidin in DMEM for 3 days. Following selection, fibroblasts were reseeded and expanded under selection. Expanded fibroblasts were then reseeded in DMEM with no selection for 24 h; following attachment, they were washed with PBS and stimulated with a dual combination of TGFβ and IL-1β (10 ng ml^−1^ each) in DMEM for 24 h. After stimulation, fibroblasts were detached with trypsin, washed in PBS and resuspended in live cell sorting buffer (PBS with 5% FBS, 25 mM HEPES, 1 mM EDTA), and the top and bottom 15% of *IL11*^*mNG*^ fibroblasts were sorted on a Sony SH800 cell sorter (see Extended Data Fig. 6a for the gating strategy). Collected fibroblasts were then spun down and frozen for subsequent DNA isolation and sequencing.

### CRISPR gDNA isolation and sequencing

Fibroblast DNA was isolated with a QIAamp DNA Micro Kit (Qiagen, catalogue no. 56304) following the manufacturer’s instructions. Isolated DNA was then eluted in water, and PCR was performed as previously described with a P5 primer and a unique P7 primer for each sample^67^. Solid-phase reversible immobilization cleanup was performed with AMPure XP beads (Beckman Coulter, A63881), and concentrations were quantified with Qubit dsDNA HS assay (Thermo Fisher Scientific, Q32854). Each sample was run on a 2% E-Gel (Thermo Fisher Scientific, G402022), the dominant bands were excised, and 4 nM of pooled DNA was sequenced with an Illumina NextSeq 500 instrument (NextSeq 500/550 High Output v.2 kit, 75 cycles) with the following parameters: single end, index 8, read 51.

### CRISPR screen analyses

PoolQ 2.2.0 (https://portals.broadinstitute.org/gpp/public/software/poolq) was used to map raw data to gRNA reads to obtain raw count data. EdgeR was used to identify differentially abundant (enriched) guides in sorted samples^68^, and STARS v.1.3 (https://portals.broadinstitute.org/gpp/public/software/stars) was used to rank the targeted genes on the basis of enrichment scores of multiple guides. Genes were selected if the perturbation was greater or equal to 3 and the fold change was greater than or equal to 2 with *P* < 0.05.

### Bulk RNA-seq of stimulated fibroblasts

*IL11*^*mNG*^ Cas9 and *IL11*^*mNG*^ Cas9–VP64 fibroblasts were infected with non-targeting lentiviral gRNA or *GLIS3* gRNA to make control, *GLIS3* CRISPRko or CRISPRa fibroblasts. Fibroblasts were expanded under 1 μg ml^−1^ puromycin and 3 μg ml^−1^ blasticidin selection to select for a bulk genetically perturbed population. *GLIS3*-perturbed fibroblasts were then seeded in DMEM. The next day, cells were washed with PBS, and new medium with or without dual TGFβ and IL-1β (10 ng ml^−1^ each) was added at 0, 6, 12 and 24 h in a reverse time course stimulation. After 24 h, fibroblasts were washed with PBS, RNA was extracted with TRIzol, and each sample’s total RNA was quantified.

mRNA was purified with a Dynabeads mRNA direct purification kit (Thermo Fisher Scientific, catalogue no. 61012) with 40 ng of total RNA per sample. RNA-seq libraries were generated with previously described preparations^69^ and sequenced with a NextSeq 500/550 high output kit v2.5 (Illumina, 20024906).

### Analysis of bulk RNA-seq of stimulated fibroblasts

Raw FASTQ reads were processed using FastQC^70^, Trim Galore (https://github.com/FelixKrueger/TrimGalore) and SortMeRNA^71^ to remove low-quality reads, adaptors and ribosomal RNA. Sequencing data were aligned to the human reference genome (GRCh38) using STAR, and gene abundance was quantified using Salmon^72^. Differentially expressed genes were identified using the DESeq2 R package^73^. Genes with *P* < 0.05 were considered to be differentially expressed.

### scRNA-seq of stimulated fibroblasts and isolated colonic mouse fibroblasts from DSS treatment

#### scRNA-seq of TGFβ- and IL-1β-stimulated fibroblasts

Fibroblasts were stimulated as for bulk RNA-seq; however, upon completion of stimulation, at least 2,000 live single cells per sample were resuspended in PBS with 0.05% BSA and submitted for 10x Genomics single-cell sequencing (3′ transcriptome V3 with Cell Suspension using a HiSeq X system for up to 300 cycles).

#### scRNA-seq of sorted PDGFRA^+^ fibroblasts

Twenty-thousand live single cells resuspended in PBS with 0.4% BSA were submitted for 10x Genomics single-cell sequencing (Chromium Next GEM Chip G, v.3.1). Libraries were sequenced across 1.2 lanes of an Illumina NovaSeqX flow cell (10B reads, up to 300 cycles).

### Analysis of scRNA-seq of stimulated fibroblasts

Gene expression matrices for each individual sample were obtained by aligning the FASTQ sequence reads to the reference hg38 human transcriptome using CellRanger v.3.1.0 (10x Genomics). Data were analysed using the scanpy package. Further, the count matrices of cell X UMI barcodes were filtered to exclude poor-quality cells, that is, those expressing fewer than 200 detected genes, more than 10,000 UMIs, or a mitochondrial gene fraction greater than 10%. Count matrices of each individual sample were merged and read-depth normalized using the standard logTP10K normalization procedure (the number of transcripts per 10,000 transcripts in a cell). The top 2,000 variable genes were then identified, and the normalized expression matrix was centred and regressed for effects of cell cycle scores (S phase and G2M phase) + UMI count per cell. Dimensionality reduction was then performed on the top 60 principal components selected on the basis of inspection of the elbow plot, followed by the construction of a *k*-nearest neighbour graph and application of the Leiden clustering algorithm. The resulting clusters were visualized in the UMAP embedding space.

### Analysis of scRNA-seq of isolated colonic mouse fibroblasts after DSS treatment

Gene expression matrices for six individual mice, three acute DSS-treated and three chronic DSS-treated, were obtained by aligning the FASTQ sequence reads to the reference mm10 mouse transcriptome using CellRanger v.7.0 (10x Genomics). One chronic DSS-treated sample failed the sequencing quality control metrics, demonstrating poor quality, and was excluded. Data were analysed using the scanpy package, following a workflow similar to that described in the ‘scRNA-seq analysis and cell type identification’ section. Altogether, we identified ten unique fibroblast cell types.

### Analysis of mouse spatial transcriptomics dataset

A gene expression matrix of 1,540,301 cells × 480 genes was obtained from colons of three wild-type DSS-treated and three *Glis3*^*f/f*^;*cre* DSS-treated mice and analysed downstream using a similar workflow to that described in the ‘Analysis of human spatial transcriptomics dataset’ section. Altogether, we identified 41 unique cell types across the different compartments.

### GLIS3^3xF^^lag^ ChIP–seq

ChIP–seq was performed according to the protocol of Tian et al.^74^ with a few modifications. Following treatment of *GLIS3*^3xF^^lag^ fibroblasts with medium alone or TGFβ and IL-1β (10 ng ml^−1^ each) for 24 h, adherent fibroblasts were washed with PBS three times, then fixed with 0.25 M disuccinimidyl glutarate (Thermo Fisher Scientific, catalogue no. 20593). Fibroblasts were washed three times in PBS, then fixed with 1% formaldehyde (MilliporeSigma, catalogue no. 1040021000). Following three more washes, fibroblasts were scraped into PBS, spun down into pellets, and resuspended in SDS lysis buffer supplemented with protease inhibitor cocktail (Sigma-Aldrich, P8340-1ML). Lysates were frozen for at least 1 h at −80 °C, followed by thawing and sonication in a microTube-500 AFA fibre screw cap (Covaris, catalogue no. 520185) on a Covaris LE220Rsc Focused Ultrasonicator-110586. Sonication was performed at 7 °C, peak incident power 450, duty factor 30%, 200 cycles per burst, treatment time 100 s, and repeated four times. Sonicated lysates were spun down and quantified for total protein and DNA using a BCA assay (Thermo Fisher Scientific, catalogue no. 23225), with measurement of absorbance at 260 nM (A260). Equal lysate concentrations were then brought up to 900 µl in low-ionic-strength chip dilution supplemented with 1 mM DTT (Thermo Fisher Scientific, R0861), and 4 µl of either IgG control (Thermo Fisher Scientific, 14-4714-82) or Flag antibody (MilliporeSigma, F1804-200UG) was added to the appropriate sample with rotation overnight at 4 °C. Following incubation, Pierce Protein G Magnetic Beads (Thermo Fisher, catalogue no. 88847) were added, followed by incubation for another 4 h at 4 °C. Beads were separated from the solution and washed twice with low-ionic-strength ChIP dilution buffer, once with high-salt buffer, once with LiCl, and finally twice with TE buffer following previously published methods^74^.

To elute from the beads, elution buffer was added for 15 min, beads were separated by magnet, and elution was repeated once more. Pooled eluates were then decross-linked overnight at constant rotation at 65 °C by addition of decross-linking buffer. Following incubation, 50 μg ml^−1^ RNase A (Qiagen, catalogue no. 19101) was added for 1 h at 65 °C; then, 0.4 mg ml^−1^ proteinase K (Thermo Fisher Scientific, AM2548) was added to digest proteins for 4 h at 65 °C. DNA was then extracted with phenol/chloroform/isoamyl alcohol (25:24:1, v/v) (Thermo Fisher Scientific, catalogue no. 15593031) extraction, and the DNA pellet was concentrated in a 2× volume of 100% ethanol, 1 μl of glycoblue coprecipitant (Thermo Fisher, AM9516) and 0.1× volume of sodium acetate (Thermo Fisher Scientific, AM9740). DNA pellets were spun down, washed in 75% ethanol, and resuspended in water. Samples then underwent library preparation for ChIP–seq using the KAPA HyperPrep Kit (Roche, catalogue no. 07962363001), following the manufacturer’s protocol.

Library yield was quantified using the dsDNA-specific assay Qubit (Thermo Fisher Scientific, Q33231). The size distribution of libraries was determined by Agilent 4200 TapeStation analysis (Agilent, G2991BA). Barcoded sample libraries at equimolar concentrations were pooled and paired-end sequenced using an Illumina NextSeq 2000 Sequencing System with NextSeq 2000 P3 Reagents Kit (200 cycles) (Illumina, catalogue no. 20040560), following the manufacturer’s instructions.

### GLIS3^3xF^^lag^ ChIP–seq analysis

ChIP–seq data were processed using the nf-core/chipseq 2.0.0 workflow^75^. Raw sequencing reads were preprocessed using FastQC and Trim Galore to remove low-quality reads and adaptors. The remaining reads were then aligned to a reference genome (GRCh38)^76^ using BWA. Picard’s MarkDuplicates (https://broadinstitute.github.io/picard/), SAMtools^77^ and BAMTools^78^ were used postalignment for filtering and removal of unmapped, multimapped, PCR duplicate and mismatched reads. BEDTools^79^ and bedGraphToBigWig^80^ were used to create normalized bigwig files, which were visualized using Integrated Genomics Viewer^81^. The retained high-quality alignment results were used to call narrow peaks using MACS2 (refs. ^82,83^) against an IgG control with *q* < 0.05. Consensus peaks set across all Flag antibody samples were created using BEDTools; featureCounts^84^ was used to count consensus peaks in each sample; and HOMER was used to annotate peaks relative to gene features and perform motif enrichment analysis.

Functional enrichment was analysed using the ClusterProfiler package^85^. Consensus annotated peaks enriched in 24-h samples were selected on the basis of having mean log_2_ fold change ≥ 0.15 relative to 0-h samples. Enriched peaks mapped to the nearest peaks were used for enrichment analysis according to the Gene Ontology Biological Processes database^86^. Pathways with Benjamini–Hochberg-adjusted *P* < 0.05 were considered for further analysis^87^.

### ChIP–qPCR

Following treatment of fibroblasts with medium alone or TGFβ and IL-1β (10 ng ml^−1^ each) for 24 h, ChIP isolation was performed as described above (‘GLIS3^3xF^^lag^ ChIP–seq’) except that 4 µg each of ChIP-grade IgG control (Thermo Fisher Scientific, catalogue no. 14-4714-82), Flag antibody (MilliporeSigma, catalogue no. F1804-200UG), FOSL1 antibody (Cell Signaling, 5841S) or TEAD1 antibody (Active Motif, catalogue no. 61643) was used. Once the DNA pellets had been resuspended in water, the samples were subjected to qPCR with iTaq Universal SYBR Green Supermix for the indicated target genes (see Supplementary Data 5 for primers). Data were normalized using the fold enrichment method, in which IP signals are divided by the IgG signals, representing the IP signal as the fold increase in signal relative to the background. Heatmaps of gene expression *z*-scores were generated in Microsoft Excel v.16.

### GLIS3 signature and PROTECT cohort analysis

Ordered probit regression, implemented using the polr function in the MASS R package^88,89^, was used to assess the association between GLIS3 signature gene expression and the clinical Mayo scores for each patient. Mayo scores were considered as the response ordinal variable, with control samples as the lowest category. The model was fitted using single-sample gene set enrichment (ssGSEA) scores and each gene’s expression independently as predictors. Bonferroni-adjusted *P* values of less than 0.05 were considered to indicate statistical significance.

### Derivation of GSEA scores

ssGSEA scores were calculated from transcriptome profiles for each subject using the ssGSEA module (v.10.1.0) implemented in GenePattern^90^.

### Deconvolution of bulk RNA-seq profiles

RNA-seq profiles for the PROTECT cohort were downloaded from GSE109142. Transcript per million data were deconvoluted to estimate the fraction of each cell type with the CIBERSORT algorithm implemented in R package RNAMagnet^91^. To define cell-type-specific markers, Wilcoxon rank-sum test was performed on the Integrated IBD Atlas. Only genes that had *P* < 1 × 10^−8^ and average log-scale fold change ≥ 0.75 compared to the background and were expressed in fewer than 20% of cells in the background population were considered further. Pseudobulk expression matrix of selected markers at cell-type-population level was used as input to CIBERSORT.

### Human participants and ethical statement

The IRB of Mass General Brigham reviewed and approved the protocol, including a waiver of informed consent from the 16 patients recruited into the PRISM cohort at MGH. The study protocol complied with all relevant ethics guidelines and regulations. Excess tissues from clinically warranted surgical resections of patients with diverticulitis, CD and UC were collected for research purposes. All samples were deidentified.

### Reporting summary

Further information on research design is available in the Nature Portfolio Reporting Summary linked to this article.

## Online content

Any methods, additional references, Nature Portfolio reporting summaries, source data, extended data, supplementary information, acknowledgements, peer review information; details of author contributions and competing interests; and statements of data and code availability are available at 10.1038/s41586-025-09907-x.

## Supplementary information

Supplementary Information A guide to supplementary data files 1–5 (data provided separately).

Reporting Summary

Supplementary Data 1 Spatial profiling patient metadata—see guide for details.

Supplementary Data 2 Bidirectional CRISPR screen hits—see guide for details.

Supplementary Data 3 List of genes selected in the custom 480-probe panels for human and mouse spatial profiling using Xenium—see guide for details.

Supplementary Data 4 Ligands used for stimulations—see guide for details.

Supplementary Data 5 Oligos used for CRISPR and qPCR—see guide for details.

## Source data

Source Data Fig. 2

Source Data Fig. 3

Source Data Fig. 4

Source Data Fig. 5

Source Data Extended Data Fig. 3

Source Data Extended Data Fig. 4

Source Data Extended Data Fig. 5

Source Data Extended Data Fig. 6

Source Data Extended Data Fig. 7

Source Data Extended Data Fig. 8

## Data Availability

Raw count matrices of the scRNA-seq data used in this study were downloaded from various repositories. Martin et al.^9^ is available at NCBI Gene Expression Omnibus (GSE134809). Smillie et al.^2^ and Kong et al.^13^ are available at the Broad Single Cell Portal (SCP259 and SCP1884, respectively). Friedrich et al.^11^ was downloaded from ImmPort (SDY1765). Processed anndata objects of scIBD are available at the Broad Single Cell Portal (SCP2927). scRNA-seq data of stimulated fibroblasts profiled at various time points are available in the NCBI Gene Expression Omnibus (GSE250516). Raw scRNA-seq data for PDGFRA^+^ fibroblasts from mouse large intestine are available in the NCBI Gene Expression Omnibus (GSE288481). Processed anndata objects of PDGFRA^+^ fibroblasts are available at the Broad Single Cell Portal (SCP3384). Bulk RNA-seq data generated in this study are available in the NCBI Gene Expression Omnibus (GSE250515). ChIP–seq data generated during this study are available in the NCBI Gene Expression Omnibus (GSE250514). CRISPR screen data generated during this study are available in Supplementary Data 2. Publicly available RNA-seq data for the PROTECT cohort were downloaded from the NCBI Gene Expression Omnibus (GSE109142). Anndata objects for Xenium-based spatial transcriptomics profiling are available in the Broad Single Cell Portal (SCP2927 for human intestinal tissue, SCP3384 for mouse intestinal tissue). Raw H&E staining images after spatial profiling are available via Zenodo at 10.5281/zenodo.17518435 (ref. ^92^). All unique biological materials generated in this study are available upon request. Source data are provided with this paper.

## Code availability

No new software or code was generated in this study. All code used for data analysis in this study is available upon request from the authors.
