# Supplementary material for: Bidirectional CRISPR screens decode a GLIS3-dependent fibrotic cell circuit
Source: Nature. 2026 Jan 7;650(8103):997–1006. doi: 10.1038/s41586-025-09907-x (PMC12820784; doi:10.1038/s41586-025-09907-x)
Supplement: Supplementary file 1 — A guide to supplementary data files 1–5 (data provided separately). [file 41586_2025_9907_MOESM1_ESM.pdf]

nature portfolio

https://doi.org/10.1038/s41586-025-09907-x

Supplementary information

Bidirectional CRISPR screens decode a GLIS3-dependent fibrotic cell circuit

In the format provided by the authors and unedited

Supplementary Data 1. Spatial profiling patient metadata. Clinical metadata for each of the patients profiled with Xenium-based spatial profiling. The data lists the PubID, diagnosis, gender, and age at diagnosis.

Supplementary Data 2. Bidirectional CRISPR screen hits. A list of CRISPR knockout and activation screen hits which control IL-11 expression ranked by a STARS score, the p-value, and fold-change enrichment. Genes highlighted in blue are enriched in both knockout and activation screens as IL-11 determinants.

Supplementary Data 3. List of genes selected in the custom 480-probe panels for human and mouse spatial profiling using Xenium. A list of gene probes used for Xenium spatial profiling of both human and mouse tissues. The gene name, Ensembl ID, and number of probes used are listed, as well as the specific cell type or condition to which this probe was used to spatially profile tissue.

Supplementary Data 4. Ligands used for stimulations. A list of cytokines or innate immune ligands used in cell stimulation assays. The agonist, final concentration used, vendor and catalog are provided.

Supplementary Data 5. Oligos used for CRISPR and qPCR. A list of oligos used for qPCR, CRISPR knockout or activation, or CRISPR knockin. Oligos are listed by gene, targeted species, sequence, and usage in the study.
