# Supplementary material for: Bidirectional CRISPR screens decode a GLIS3-dependent fibrotic cell circuit
Source: Nature. 2026 Jan 7;650(8103):997–1006. doi: 10.1038/s41586-025-09907-x (PMC12820784; doi:10.1038/s41586-025-09907-x)
Supplement: Supplementary file 2 — Reporting Summary [file 41586_2025_9907_MOESM2_ESM.pdf]

nature portfolio

Corresponding author(s): Ramnik Xavier, Daniel Graham

Last updated by author(s): Oct 28, 2025

Reporting Summary

Nature Portfolio wishes to improve the reproducibility of the work that we publish. This form provides structure for consistency and transparency in reporting. For further information on Nature Portfolio policies, see our Editorial Policies and the Editorial Policy Checklist.

Statistics

For all statistical analyses, confirm that the following items are present in the figure legend, table legend, main text, or Methods section.

n/a	Confirmed	
☐	☒	The exact sample size (*n*) for each experimental group/condition, given as a discrete number and unit of measurement
☐	☒	A statement on whether measurements were taken from distinct samples or whether the same sample was measured repeatedly
☐	☒	The statistical test(s) used AND whether they are one- or two-sided<br>*Only common tests should be described solely by name; describe more complex techniques in the Methods section.*
☐	☒	A description of all covariates tested
☒	☐	A description of any assumptions or corrections, such as tests of normality and adjustment for multiple comparisons
☐	☒	A full description of the statistical parameters including central tendency (e.g. means) or other basic estimates (e.g. regression coefficient) AND variation (e.g. standard deviation) or associated estimates of uncertainty (e.g. confidence intervals)
☐	☒	For null hypothesis testing, the test statistic (e.g. *F*, *t*, *r*) with confidence intervals, effect sizes, degrees of freedom and *P* value noted<br>*Give P values as exact values whenever suitable.*
☐	☒	For Bayesian analysis, information on the choice of priors and Markov chain Monte Carlo settings
☒	☐	For hierarchical and complex designs, identification of the appropriate level for tests and full reporting of outcomes
☒	☐	Estimates of effect sizes (e.g. Cohen's *d*, Pearson's *r*), indicating how they were calculated

Our web collection on statistics for biologists contains articles on many of the points above.

Software and code

Policy information about availability of computer code

Data collection	No software was used
Data analysis	Ingenuity Pathway Analysis, FlowJo v10.8, GraphPad PRISM v10, Fiji ImageJ v1.54p, Microsoft Excel v16, QuPath v0.6.0, CellRanger v3.1.0

For manuscripts utilizing custom algorithms or software that are central to the research but not yet described in published literature, software must be made available to editors and reviewers. We strongly encourage code deposition in a community repository (e.g. GitHub). See the Nature Portfolio guidelines for submitting code & software for further information.

Data

Policy information about availability of data

All manuscripts must include a data availability statement. This statement should provide the following information, where applicable:

- Accession codes, unique identifiers, or web links for publicly available datasets
- A description of any restrictions on data availability
- For clinical datasets or third party data, please ensure that the statement adheres to our policy

Raw count matrices of the single-cell RNA-sequencing data used in this study were downloaded from various repositories. Martin et al. 2019 is available at NCBI Gene Expression Omnibus (GSE134809). Smillie et al. 2019 and Kong et al. 2023 are available at the Broad Single Cell Portal (SCP259 and SCP1884, respectively). Friedrich et al. 2021 was downloaded from ImmPort (SDY1765). Processed anndata objects of single cell RNAseq IBD atlas are available at the Broad Single Cell Portal (SCP2927).

Single-cell RNA-sequencing data of stimulated fibroblasts profiled at various time points are available in the NCBI Gene Expression Omnibus (GSE250516). Raw single-cell RNA-sequencing data for PDGFRA+ fibroblasts from the mouse large intestine are available in the NCBI Gene Expression Omnibus (GSE288481). Processed anndata object of PDGFRA+ fibroblasts is available at the Broad Single Cell Portal (SCP3384). Bulk RNA-sequencing data generated in this study are available in the NCBI Gene Expression Omnibus (GSE250515). ChIP-seq data generated during this study are available in the NCBI Gene Expression Omnibus (GSE250514). CRISPR screen data generated during this study are available in Supplementary Data 2. Publicly available RNA-sequencing data for the PROTECT cohort was downloaded from the NCBI Gene Expression Omnibus (GSE109142). Anndata objects of Xenium based spatial transcriptomics profiling is available in the Broad Single Cell Portal (SCP2927 for human intestinal tissue, SCP3384 for mouse intestinal tissue). Raw Hematoxylin and eosin (H&E) stained images post-spatial profiling are available on Zenodo at https://doi.org/10.5281/zenodo.17518435.

Research involving human participants, their data, or biological material

Policy information about studies with human participants or human data. See also policy information about sex, gender (identity/presentation), and sexual orientation and race, ethnicity and racism.

Reporting on sex and gender	We did not report on sex and gender differences in our datsets as this analysis is not applicable to the scope of our study. We provide self-reported genders of human patients. Consent has been provided.
Reporting on race, ethnicity, or other socially relevant groupings	We did not report on race, ethnicity or other groupings in our datasets as this analysis is not applicable to the scope of our study.
Population characteristics	For single-cell studies, information is provided in each separate previously-published studies' methods. For spatial-sequencing studies, 16 patients diagnosed with ulcerative colitis (UC), Crohn's disease (CD), or diverticulitis (DC) were profiled. These patients varied in age, gender, and age of disease diagnosis. Patient tissues ranged on a disease spectrum, from non-inflamed and normal, to different grades of inflammation, ulceration, fibrosis, and the presence or absence of fibromuscular hyperplasia.
Recruitment	For single-cell studies, information is provided in each separate previously-published studies' methods.<br>For spatial studies, 16 patients diagnosed with ulcerative colitis (UC), Crohn's disease (CD), or diverticulitis (DC) were recruited into the Prospective Registry in IBD Study at MGH (PRISM) study at Massachusetts General Hospital (MGH).
Ethics oversight	Intestinal tissue spatial profiling was approved by the Mass General Brigham IRB.

Note that full information on the approval of the study protocol must also be provided in the manuscript.

Field-specific reporting

Please select the one below that is the best fit for your research. If you are not sure, read the appropriate sections before making your selection.

☒ Life sciences ☐ Behavioural & social sciences ☐ Ecological, evolutionary & environmental sciences

For a reference copy of the document with all sections, see nature.com/documents/nr-reporting-summary-flat.pdf

Life sciences study design

All studies must disclose on these points even when the disclosure is negative.

Sample size	For mice, the minimal sample size was set the availability of mice for completing the experiment, with each treatment or genotype consisting of at least n=3. Inbred mice were used and sex-matched, allowing for similar variance between experimental group. In vitro cell culture experiments were performed with at least 3 independent biological replicates for each experiment. This is consistent with previous studies and accounts for biological variability between distinct samples from cell lines. Although statistical methods were not used to calculate sample size, we used a minimum of 3 biological replicates.
Data exclusions	No data were excluded from analyses.
Replication	All in vitro experiments consisted of at least 3 independent biological replicates and were repeated at least twice in independent experiments, and all replication attempts were successful. For mouse experiments, all experiments consisted of at least 3 animals per genotype/condition, and were repeated 2 to 4 times in independent experiments.
Randomization	For mouse experiments, genotype-matched mice were randomly assigned to each treatment group. For in vitro experiments, randomization was not applicable, as experiments consisted of predefined experimental layouts stratified by genotype and treatment into independent biological replicates.
Blinding	Experiments influenced by observer bias were avoided by a blinded evaluation by a third-party of de-identified tissue sections from either human or mouse. Blinding was not technically applicable to other experiments.

Reporting for specific materials, systems and methods

We require information from authors about some types of materials, experimental systems and methods used in many studies. Here, indicate whether each material, system or method listed is relevant to your study. If you are not sure if a list item applies to your research, read the appropriate section before selecting a response.

Materials & experimental systems

n/a	Involved in the study
☐	☒ Antibodies
☐	☒ Eukaryotic cell lines
☒	☐ Palaeontology and archaeology
☐	☒ Animals and other organisms
☒	☐ Clinical data
☒	☐ Dual use research of concern
☒	☐ Plants

Methods

n/a	Involved in the study
☐	☒ ChIP-seq
☐	☒ Flow cytometry
☒	☐ MRI-based neuroimaging

Antibodies

Antibodies used

Mouse IgG1 kappa Isotype Control (Thermo Fisher #14-4714-82), anti-mNeonGreen (Proteintech #nfms), anti-CD68 (Cell Signaling #97778S), anti-FLAG (MilliporeSigma F1804-200UG), anti-Collagen 6 (Thermo Fisher #PA5-106556), anti-Fosl1 (Cell Signaling, #5841S), anti-TEAD1 (Active Motif, #61643), anti-IgG (BioXCell, #BP0083), anti-TGFβ (BioXCell, #BP0057), anti-IL-1β (Invivogen, #mil1b-mab9-1T), AF-488 conjugated anti-mouse (Proteintech #sms1AF488-1), AF-594 conjugated anti-rabbit (ThermoFisher #A-21207), AF-647 conjugated anti-mouse (Thermo Fisher #A-21235).

Validation

Antibody validation statements or validation data for each applicable assay were provided on the manufacturer's websites, including references to prior publications utilizing these antibodies in the applicable assay.
Mouse IgG1 kappa Isotype Control: https://www.thermofisher.com/order/genome-database/dataSheetPdf?producttype=antibody&productsubtype=antibody_control&productId=14-4714-82&version=Local
anti-mNeonGreen: PMID: 40176062
anti-CD68: https://www.cellsignal.com/products/primary-antibodies/cd68-e3o7v-rabbit-mab/97778?index=1&application=IF
anti-FLAG: PMID: 32728244
anti-Collagen 6: https://www.thermofisher.com/antibody/product/COL6A2-Antibody-Polyclonal/PA5-106556
anti-Fosl1: https://www.cellsignal.com/products/primary-antibodies/phospho-fra1-ser265-d22b1-rabbit-mab/5841/applications?index=1&application=ChIP&type=pdp
anti-TEAD1: https://www.activemotif.com/catalog/details/61643/tead1-antibody-pab
anti-IgG: PMID: 29669251
anti-TGFβ: PMID: 37236193
anti-IL-1β: https://www.invivogen.com/recombinant-anti-mouse-il1beta-antibody
AF-488 conjugated anti-mouse: https://www.ptglab.com/products/pictures/pdf/sms1AF488-1_Datasheet_Alpaca_anti-mouse_IgG1__Fc-specific_recombinant_VHH__Alexa_Fluor__488_190924.pdf
AF-594 conjugated anti-rabbit: https://www.thermofisher.com/antibody/product/Donkey-anti-Rabbit-IgG-H-L-Highly-Cross-Adsorbed-Secondary-Antibody-Polyclonal/A-21207
AF-647 conjugated anti-mouse: https://www.thermofisher.com/antibody/product/Goat-anti-Mouse-IgG-H-L-Cross-Adsorbed-Secondary-Antibody-Polyclonal/A-21235

Eukaryotic cell lines

Policy information about cell lines and Sex and Gender in Research

Cell line source(s)

Primary colon fibroblasts (ATCC, CRL-1459) (female), and immortalized fibroblasts (ATCC, CRL-4001) (male) were obtained from ATCC. Lenti-X 293T cells were obtained from Takara (#632180) (female). Primary monocyte-derived macrophages were from isolated from healthy donor blood (unpurified buffy coats, 25-50mL) (ages within 18-65 years, unknown distribution of male and female) were collected at Research Blood Components, LCC, MA, USA after obtaining a signed consent form. THP-1-Cas9-expressing cells were a gift from the Genomics Platform of Broad Institute of MIT and Harvard (male).

Authentication

Commercially-purchased cell lines were authenticated by vendor ATCC with short tandem repeat identification. Lenti-X 293T cells from Takara are a subcloned HEK cell line which produces high transfectability and virus production, and details regarding authentication were not provided by the manufacturer. Donor or donated cells were not authenticated.

Mycoplasma contamination

All cell lines tested negative for mycoplasma, except donor-derived cells which were not tested.

Commonly misidentified lines
(See ICLAC register)

CNDT2 cells can be misidentified as 293 cells (https://iclac.org/databases/cross-contaminations), although the former was not used in this study. Lenti-X 293T cells were only utilized for production of lentivirus in this study, and no assays were performed in this cell line.

Animals and other research organisms

Policy information about studies involving animals; ARRIVE guidelines recommended for reporting animal research, and Sex and Gender in Research

Laboratory animals	Male and female C57BL/6J mice, aged 5 to 20 weeks old. Mice were housed with a 12 hour dark, 12 hour light cycle and provided food and water ad libitum. Mice were housed at an ambient temperature of 18–24°C and 30–70% relative humidity.
Wild animals	No wild animals used.
Reporting on sex	Sex was considered in the study design. For each experiment, age- and sex-matched mice were used.
Field-collected samples	No field collected samples.
Ethics oversight	All animal procedures were conducted in accordance with protocol 2003N000158 approved by the Massachusetts General Hospital Institutional Animal Care and Use Committee (IACUC), and animals were cared for according to the requirements of the National Research Council's Guide for the Care and Use of Laboratory Animals.

Note that full information on the approval of the study protocol must also be provided in the manuscript.

Plants

Seed stocks	Not applicable.
Novel plant genotypes	Not applicable.
Authentication	Not applicable.

ChIP-seq

Data deposition

☒ Confirm that both raw and final processed data have been deposited in a public database such as GEO.

☒ Confirm that you have deposited or provided access to graph files (e.g. BED files) for the called peaks.

Data access links<br>*May remain private before publication.*	https://www.ncbi.nlm.nih.gov/geo/query/acc.cgi?acc=GSE250514<br>token - ancreuuqbdkndmv
Files in database submission	IgG.bigWig<br>FLAG_0hr_REP4.bigWig<br>FLAG_24hr_REP1.bigWig<br>FLAG_0hr_REP2.bigWig<br>FLAG_0hr_REP3.bigWig<br>FLAG_24hr_REP2.bigWig<br>FLAG_0hr_REP1.bigWig<br>FLAG_24hr_REP4.bigWig<br>FLAG_0hr_REP5.bigWig<br>FLAG_24hr_REP3.bigWig<br>FLAG.consensus_peaks.featureCounts.txt<br>FLAG.consensus_peaks.annotatePeaks.txt<br>FLAG.consensus_peaks.bed<br>FLAG.consensus_peaks.boolean.annotatePeaks.txt
Genome browser session<br>(e.g. UCSC)	*Provide a link to an anonymized genome browser session for "Initial submission" and "Revised version" documents only, to enable peer review. Write "no longer applicable" for "Final submission" documents.*

Methodology

Replicates	4-5 independent biological replicates were used per condition.
Sequencing depth	Filename,Total Sequences,total_deduplicated_percentage,Sequence length,single or paired-end<br>FLAG_0hr_REP1_T1_1.fastq.gz,63825497,85.12147890380585,100,paired-end

FLAG_0hr_REP1_T1_2.fastq.gz,63825497,85.98601570941081,100,paired-end
FLAG_0hr_REP2_T1_1.fastq.gz,65783002,84.13266896151427,100,paired-end
FLAG_0hr_REP2_T1_2.fastq.gz,65783002,85.0906634142619,100,paired-end
FLAG_0hr_REP3_T1_1.fastq.gz,67071532,85.9378035485285,100,paired-end
FLAG_0hr_REP3_T1_2.fastq.gz,67071532,86.59984451113537,100,paired-end
FLAG_0hr_REP4_T1_1.fastq.gz,68097560,85.64251650881837,100,paired-end
FLAG_0hr_REP4_T1_2.fastq.gz,68097560,86.35781558601163,100,paired-end
FLAG_0hr_REP5_T1_1.fastq.gz,65255142,86.66659921680682,100,paired-end
FLAG_0hr_REP5_T1_2.fastq.gz,65255142,87.38212332610901,100,paired-end
FLAG_24hr_REP1_T1_1.fastq.gz,48039800,85.69879275105889,100,paired-end
FLAG_24hr_REP1_T1_2.fastq.gz,48039800,86.43442704073722,100,paired-end
FLAG_24hr_REP2_T1_1.fastq.gz,68013761,85.79277080080696,100,paired-end
FLAG_24hr_REP2_T1_2.fastq.gz,68013761,86.48711026620319,100,paired-end
FLAG_24hr_REP3_T1_1.fastq.gz,54187844,86.242582870687,100,paired-end
FLAG_24hr_REP3_T1_2.fastq.gz,54187844,87.18195284798223,100,paired-end
FLAG_24hr_REP4_T1_1.fastq.gz,50719575,85.15964084543576,100,paired-end
FLAG_24hr_REP4_T1_2.fastq.gz,50719575,86.89863705769343,100,paired-end
IgG_REP1_T1_1.fastq.gz,58694203,84.65580009741987,100,paired-end
IgG_REP1_T1_2.fastq.gz,58694203,85.59474324846335,100,paired-end
IgG_REP1_T2_1.fastq.gz,69215397,84.4831019945503,100,paired-end
IgG_REP1_T2_2.fastq.gz,69215397,85.2129231532157,100,paired-end
IgG_REP1_T3_1.fastq.gz,69917781,84.84554124011555,100,paired-end
IgG_REP1_T3_2.fastq.gz,69917781,85.74468687409727,100,paired-end
IgG_REP1_T4_1.fastq.gz,65788438,83.63129547810495,100,paired-end
IgG_REP1_T4_2.fastq.gz,65788438,84.69134057534067,100,paired-end

Antibodies

anti-FLAG (MilliporeSigma F1804-200UG), mouse IgG1 kappa Isotype Control (Thermo Fisher #14-4714-82)

Peak calling parameters

ChIP-seq data were processed using the nf-core/chipseq 2.0.0 workflow (Ewels et al. 2020). Raw sequencing reads were preprocessed using FastQC and Trim Galore! to remove low-quality reads and adapters. The remaining reads were then aligned to a reference genome (GRCh38)(Schneider et al. 2017) using BWA. Picard's MarkDuplicates (https://broadinstitute.github.io/picard/), SAMtools(Danecek et al. 2021), and BAMTools(Barnett et al. 2011) were used post-alignment for filtering and removing unmapped, multi-mapped, PCR duplicate, and mismatched reads. BEDTools(Quinlan and Hall 2010) and bedGraphToBigWig(Kent et al. 2010) were used to create normalized bigwig files which were visualized using Integrated Genomics Viewer (IGV). The retained high-quality alignment results were used to call narrow peaks using Model-based Analysis of ChIP-seq version 2 (MACS2)(Zhang et al. 2008; Gaspar 2018) against an IgG control with a q-value < 0.05.

Data quality

Sample,count of peaks with q-value<0.05
FLAG_0hr_REP1,25
FLAG_0hr_REP2,49
FLAG_0hr_REP3,46
FLAG_0hr_REP4,20
FLAG_0hr_REP5,15
FLAG_24hr_REP1,25
FLAG_24hr_REP2,98
FLAG_24hr_REP3,618
FLAG_24hr_REP4,769

Software

Chipseeker, clusterProfiler, DESeq2

Flow Cytometry

Plots

Confirm that:

☒ The axis labels state the marker and fluorochrome used (e.g. CD4-FITC).

☒ The axis scales are clearly visible. Include numbers along axes only for bottom left plot of group (a 'group' is an analysis of identical markers).

☒ All plots are contour plots with outliers or pseudocolor plots.

☒ A numerical value for number of cells or percentage (with statistics) is provided.

Methodology

Sample preparation

For in vitro fibroblasts, cells were trypsinized, washed with PBS, and re-suspended in cold live-cell sorting buffer (PBS with 5% FBS, 2mM EDTA, 25mM HEPES).

For whole-colon tissue, colon was processed by following the methods of Morral C. et al. Isolation of Epithelial and Stromal Cells from Colon Tissues in Homeostasis and Under Inflammatory Conditions. Bio Protoc. 2023 Sep 20;13(18):e4825. doi: 10.21769/BioProtoc.4825. PMID: 37753470.

Instrument

Sony SH800 cell sorter, Beckman CytoFLEX S cytometer, Beckman CytoFLEX LX cytometer

Software | Flowjo

Cell population abundance | For in vitro fibroblasts, no population was sorted from a multi-cellular source. Fibroblast population purity was maintained using ultra-purity sorting, followed by confirming sorted cells through Sony SH800 cell sorter.

For colons, abundance was measured by manual cell counting-post sort. Purity was maintained using ultra-purity sorting, followed by detailed characterization of isolated cell types with scRNA-seq.

Gating strategy | Single population of fibroblasts was used for flow cytometry. Bulk cell population was gated away from small debris using FSC/SSC. Doubles were excluded by gating on FSC-H/FSC-A. FITC-A was used as measurement of IL-11mNeonGreen median fluorescence intensity.

For colons, gating was done as follows. All cells were first gated by FSC-A/SSC-A, followed by exclusion of dead cells by gating live cells on Live/Dead stain on APC-AF50-A. PECAM1 and TER119 cells were excluded by drawing a gate on PE-A and PE-Dazzle-A negative cells, respectively. CD45 and EPCAM1 cells were excluded by drawing a gate on APC-A and BV711-A negative cells, respectively. PDGFRA+, IL-11mNG cells were then gated. IL-11mNG+ cells were assessed by comparing to signal from water-treated mice.

☒ Tick this box to confirm that a figure exemplifying the gating strategy is provided in the Supplementary Information.
